# Fluorescent Albumin-Binding *N*-Propylbenzene Indolenine-Based Squaraines as Potential Candidates for Prostate Cancer Photodynamic Therapy Photosensitizers

**DOI:** 10.3390/ijms262210989

**Published:** 2025-11-13

**Authors:** Catarina Costa, Eurico Lima, Maria Vaz, Octávio Ferreira, Renato E. Boto, Paulo Almeida, José R. Fernandes, Samuel M. Silvestre, Lucinda V. Reis

**Affiliations:** 1 CQ-VR—Chemistry Centre of Vila Real, University of Trás-os-Montes and Alto Douro, Quinta de Prados, 5001-801 Vila Real, Portugal; 2 RISE-Health, Faculty of Health Sciences, University of Beira Interior, Avenida Infante D. Henrique, 6201-506 Covilhã, Portugal; 3 RISE-Health, Faculty of Sciences, University of Beira Interior, Rua Marquês d’Ávila e Bolama, 6201-001 Covilhã, Portugal

**Keywords:** squaraine dyes, fluorescent probes, human serum albumin, photosensitizer

## Abstract

Squaraine dyes are a class of organic compounds that exhibit some characteristics inherent to those of an “ideal photosensitizer”, such as high absorption at near-infrared-close wavelengths and to produce reactive oxygen species. The introduction of amines into their squaric ring, although known to increase the phototoxicity of squaraines, can improve dyes’ water solubility and induce bathochromic shifts compared to their unsubstituted derivatives, interesting effects in biological contexts. In this work, four new squaraines were synthesized and structurally, photophysically, and photochemically characterized (including absorption and aggregation, fluorescence, light stability, and singlet oxygen generation). Their potential as fluorescent probes for albumin detection was assessed through both in silico and in vitro approaches, as well as their suitability as potential photosensitizers for photodynamic therapy. For this last purpose, the 663 nm light-induced effects of the new dyes were evaluated against the PC-3 prostate cancer cell line, while their photocytotoxicity toward normal human dermal fibroblasts was also assessed using the MTT assay, to determine their potential tumor-selective effects. Low singlet oxygen quantum yields suggest that type I reactions predominate in generating cytotoxicity. Overall, the findings indicate that the designed squaraines exhibit moderate yet favorable interactions with albumin protein while demonstrating selective photodynamic effects toward prostate adenocarcinoma cancer cells, highlighting their potential as protein-assisted, tumor-targeted photosensitizers, providing a basis for further mechanistic studies.

## 1. Introduction

Photodynamic therapy (PDT) is a minimally invasive treatment modality with great promise due to its selective action on specific cell types and its low incidence of adverse effects. PDT involves the administration of a photosensitizer that, upon irradiation with light of a suitable wavelength in the presence of ground-state triplet oxygen, generates reactive oxygen species (ROS), which, although essential for numerous vital cellular functions, induce cytotoxicity when produced in excessive amounts [1,2,3].

To date, the range of photosensitizers approved for clinical use remains limited, with the vast majority being porphyrin derivatives or related analogues [4]. The identification of new candidates with optimal properties for therapeutic application is therefore crucial. For a molecule to serve effectively as a photosensitizer, several requirements must be met: strong absorption (>10^5^ M^−1^ cm^−1^) within the “phototherapeutic window” (600–850 nm), where reduced tissue scattering allows deeper light penetration [5]; safety in the absence of light; efficient generation of ROS upon light activation; preferential accumulation in the target tissue; and, ideally, suitability for administration through multiple routes [6,7].

Squaraine dyes, a distinctive class of non-porphyrinic organic compounds, consisting of 1,3-disubstituted derivatives of 3,4-dihydroxycyclobut-3-ene-1,2-dione (commonly known as squaric acid) first reported by Treibs and Jacob in 1965 [8], are characterized by a highly planar, extensively conjugated system centered on a four-membered squaric ring, symmetrically flanked by electron-donating groups. This unique electronic structure endows squaraines with noteworthy optical and electronic properties, making them attractive for a wide range of technological and biomedical applications [9]. Reported uses include fluorescent probes [10,11,12,13], active materials for organic light-emitting diodes [14,15], sensitizers for organic solar cells [16,17,18,19], ligands for G-quadruplex structures [20,21], and promising candidates as photosensitizers in PDT [22,23,24,25].

Interestingly, regarding their potential use in PDT, several cyanines and squaraines have been synthesized in recent years and have shown promise as photosensitizers for cancer treatment due to their favorable photophysical and photochemical properties that align well with the requirements of PDT [26,27,28]. Nevertheless, much remains to be understood about this class of compounds, particularly in establishing clear structure–activity relationships [29]. Moreover, depending on the structural modifications introduced into their scaffold, squaraines may display additional advantageous features, including high photochemical stability and moderate fluorescence quantum yields, along with enhanced fluorescence intensity upon non-covalent binding to specific ligands [10,30,31,32,33]. These attributes render squaraine dyes valuable fluorescent probes for protein detection, as their fluorescence can be selectively activated in the presence of target proteins.

The most abundant protein in the human circulatory system is human serum albumin (HSA), which has been extensively studied due to its high bioavailability, low cost, remarkable stability, and ability to transport a wide range of ligands to specific sites [12]. This protein plays a crucial role in maintaining metabolic processes, including the regulation of plasma oncotic pressure, the reduction in toxin activity, and the control of plasma antioxidant properties [34,35]. Its importance stems from its capacity to transport a variety of macromolecules, such as hormones, lipids, metal ions, amino acids, and drugs [36]. Moreover, HSA is a key biomarker for evaluating a patient’s health status, since fluctuations in its concentration are often associated with diseases such as cardiovascular disorders, liver dysfunction, and nephrotic syndrome [12,37]. Consequently, the development of more sensitive methodologies for its detection and quantification is of great relevance in fields such as biochemistry, biotechnology, and immunodiagnostics [38].

In this work, we report on the synthesis and structural, photophysical, and photochemical characterization of *N*-propylbenzene indolenine-based squaraines, aiming to explore their potential as fluorescent probes for the detection and quantification of HSA. The in silico and in vitro studies were performed specifically to evaluate their suitability as probes, while *in cell* experiments were conducted to assess their photobiological properties, particularly regarding their potential as photosensitizers in PDT. Additionally, at the interface of these studies, we investigated the possible role of albumin in mediating the systemic transport of these dyes upon prospective administration.

## 2. Results

### 2.1. Chemistry

**Figure 1:**
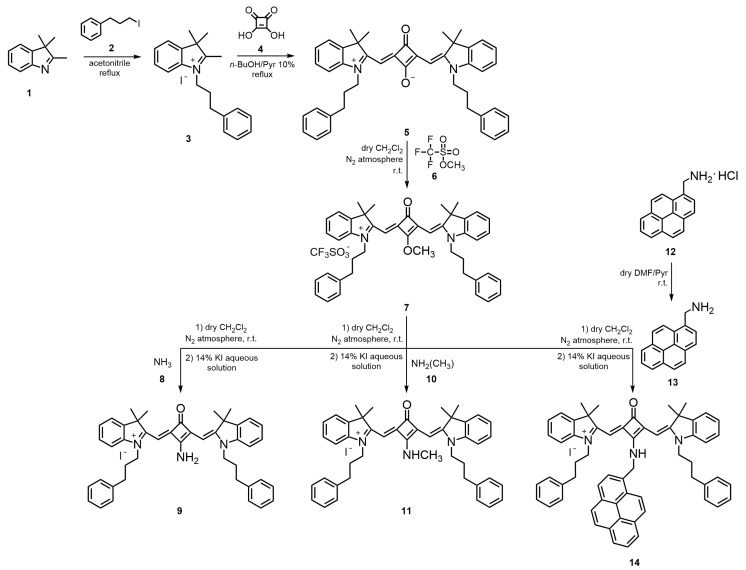
Synthetic route of *N*-propylbenzene indolenine-based squaraine dyes **5**, **7**, **9**, **11**, and **14**.

The *N*-propylbenzene chain indolenine-based squaraine dyes **5**, **7**, **9**, **11**, and **14** were synthesized through a multistep approach, as outlined in Figure 1. To the best of our knowledge, both the complete structural characterization, together with their photophysical and photochemical properties, are reported here for the first time.

The synthesis began with the *N*-alkylation of 2,3,3-trimethylindolenine (**1**) using an excess of 3-iodo-1-phenylpropane (**2**) in refluxing acetonitrile for six days, affording the corresponding quaternary ammonium salt **3** in 79% yield. Subsequent condensation of salt **3** with 0.5 equivalents of squaric acid (**4**) in a mixture of *n*-butanol/pyridine (10%) under reflux for 7 h gave the unsubstituted zwitterionic squaraine dye **5** in 85% yield. From this intermediate, the *O*-methylated derivative **7** was prepared by the reaction of dye **5** with three molar equivalents of methyl trifluoromethanesulfonate (**6**) in dry dichloromethane, under nitrogen at room temperature, for 3 h. This derivative served as the key precursor for the preparation of amino-substituted squaraine dyes. The displacement of the methoxy group in *O*-methylated dye **7** with amines **8**, **10**, and **13** furnished the corresponding *N*-propylbenzene aminosquaraines **9**, **11**, and **14**, respectively, in moderate yields (22–43%). For the synthesis of dye **14**, the amine **13** was first obtained by neutralization of its hydrochloride salt precursor **12** with pyridine in dry dimethylformamide (DMF) at room temperature, providing the free base suitable for nucleophilic substitution. Finally, all aminosquaraine products were subjected to counterion exchange by treatment with a 14% aqueous potassium iodide solution, yielding the iodide salts.

On the infrared (IR) spectra, weak bands characteristic of aromatic C–H bonds appeared in the region between 3102 and 3024 cm^−1^, and slightly stronger bands related to the vibrations of aliphatic C–H bonds between 2970 and 2860 cm^−1^. In the case of aminosquaraine dyes **9**, **11**, and **14**, weak vibrations of the NH bonds appeared between 3256 and 3088 cm^−1^. It would be expected that a characteristic band of C=O in the four-membered central ring would be observed at approximately 1700 cm^−1^. However, this band only appeared in the spectrum of squaraine dye **7**, which is justified by the fact that there is resonance between the nitrogen atom and oxygen atom of the carbonyl group of the other dyes. Around 1600 cm^−1^, strong bands of C=C bonds appeared in all squaraines.

**Figure 2:**
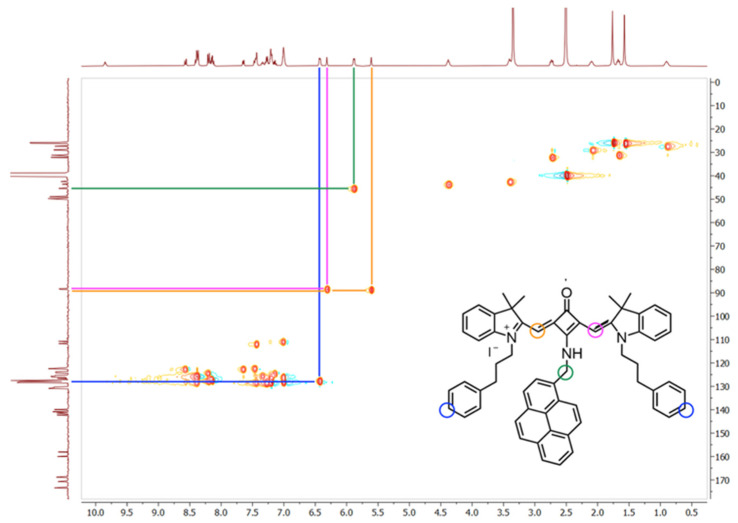
Heteronuclear single quantum coherence spectroscopy spectrum of aminomethylpyrene-bearing *N*-propylbenzene indolenine-based squaraine dye **14**. The different colors of the lines indicate correlations between specific protons and carbons in the squaraine dye.

Regarding the most relevant signals (Figures S1–S15), in the ^1^H NMR spectrum of squaraine **9**, a singlet of two protons at 9.07 ppm was observed, assigned to the protons of the NH_2_ group, which disappeared upon D_2_O exchange. For dye **11**, a single-proton broad singlet at 9.06 ppm corresponded to the proton of the methylamino group, while a three-proton singlet at 3.27 ppm was attributed to the methyl substituent, further supported in the ^13^C NMR spectrum by a new signal at 30.46 ppm. For squaraine **14**, in the ^13^C NMR spectrum, a distinct signal at 43.61 ppm corresponded to the methylene carbon of the NHCH_2_ group, which was further evidenced in the ^1^H NMR spectrum by additional resonances characteristic of the 1-aminomethylpyrene moiety: the NHCH_2_–pyrene proton appeared as a single proton triplet at 9.85 ppm, disappearing upon D_2_O exchange, while the methylene protons resonated as a two protons doublet at 5.88 ppm (green correspondence; Figure 2), collapsing to a singlet after D_2_O addition. A multiplet at 6.44–6.42 ppm was assigned to two aromatic protons of the benzene units of *N*-propylbenzene chains, confirmed by heteronuclear single quantum coherence spectroscopy (blue correspondence).

Squaraine dyes **5**, **7**, and **9** were shown to be symmetrical, so their spectra displayed only half of the squaraine-core proton and carbon signals. In contrast, as expected, squaraine dye **11** lost symmetry upon the introduction of a methylamino substituent into the squaric ring, whereas the asymmetry of dye **14** likely arose from the partial double-bond character between the central ring carbon and the nitrogen atom. This effect, highlighted, for instance, by the signals corresponding to the methine protons and carbons marked in orange and pink (Figure 2), restricts the rotation of the C–NH groups of these molecules.

### 2.2. Photochemical and Photophysical Characterization

Determining the spectral region in which these molecules absorb, as well as the intensity of that absorption, is highly relevant for their photobiological applications. Ideally, photosensitizers intended for PDT should absorb within the so-called “phototherapeutic window” (600–850 nm), where most endogenous biomolecules do not absorb energy. Furthermore, from the perspective of their use as fluorescent probes, it is important to understand their absorption profile to predict the corresponding emission behavior.

Accordingly, photophysical studies were carried out in three organic solvents (chloroform, methanol, and dimethyl sulfoxide, in order of increasing polarity; Table 1). The dyes showed absorption within the ideal spectral range (λ_abs_ = 631–663 nm), accompanied by high molar absorption coefficients (ε = 1.74–4.28 × 10^5^ M^−1^·cm^−1^). The absorption behavior observed in the three solvents cannot be explained solely based on solvent polarity, as the trend did not follow a classical solvatochromic pattern. In methanol, the hypsochromic shift can be attributed to hydrogen bonding between the solvent and the dye, which preferentially stabilizes the ground state over the excited state. In contrast, dimethyl sulfoxide, a polar aprotic solvent, efficiently stabilizes charge-separated excited states through dipole–dipole interactions, leading to a bathochromic shift. Chloroform, being weakly polar and lacking hydrogen-bond donor ability, provides only moderate stabilization of either state, resulting in absorption maxima that lie between those recorded in methanol and dimethyl sulfoxide. Overall, these results highlight the importance of dye–solvent interactions, particularly hydrogen bonding and dipolar stabilization, rather than polarity alone, in dictating the photophysical properties of the dyes.

In terms of fluorescence performance, the squaraine dyes exhibited solvent-dependent behavior. Among them, the unsubstituted squaraine dye **5** showed the highest fluorescence quantum yield, reaching Φ_F_ = 1.00 in dimethyl sulfoxide, which clearly surpassed the substituted derivatives. The introduction of amine groups did not enhance this property; in fact, methylamino- and aminomethylpyrene-bearing dyes **11** and **14** displayed markedly lower quantum yields, with values of 0.04 and 0.05 in dimethyl sulfoxide, respectively. Dye **9**, also substituted with an amine, reached only Φ_F_ = 0.12 in dimethyl sulfoxide. Regardless of solvent, all aminosquaraines maintained only modest fluorescence efficiency, with yields further decreasing in chloroform and methanol (Φ_F_ = 0.02–0.08).

Despite these limitations, the dyes consistently exhibited moderate Stokes shifts (ΔS = 9–15 nm), a feature that supports their potential applicability as fluorescent probes, where spectral separation between the absorption and emission bands is desirable.

Squaraine dyes, like other extended π-conjugated scaffold chromophores, exhibit a pronounced tendency to aggregate in aqueous media due to their high planarity and hydrophobic character. In these environments, π–π stacking interactions promote the formation of hypsochromic absorption bands, called H-aggregates, or J-aggregates, that originate bathochromic bands, depending on the relative orientation of the dye molecules.

**Figure 3:**
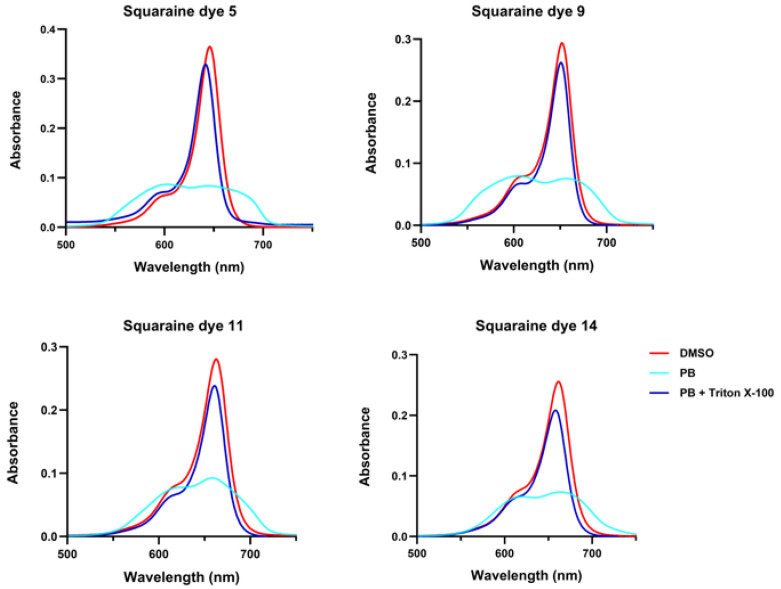
Aggregation behavior of *N*-propylbenzene indolenine-based squaraine dyes **5**, **9**, **11**, and **14**. Vis absorption spectra were recorded in dimethyl sulfoxide (DMSO, red), phosphate buffer (PB, cyan), and phosphate buffer with Triton X-100 at a 1% final concentration (PB + Triton X-100, blue).

For the synthesized squaraines, it was observed that in phosphate buffer, they tended to aggregate regardless of their molecular structure, which drastically reduced their absorption capacity and led to a pronounced broadening of the absorption band (Figure 3). This band broadening is a clear indication of aggregate formation. Squaraines **5**, **9**, and **11** displayed both hypsochromic and bathochromic components compared to their spectra in dimethyl sulfoxide, confirming the coexistence of H- and J-type aggregates. In contrast, the pyrene-containing dye **14** appeared to predominantly form J-aggregates, as suggested by the stronger red-shifted features observed visually. Upon the addition of Triton X-100 detergent, the aggregates were disrupted, and the absorption spectra of all dyes closely resembled those recorded in dimethyl sulfoxide, demonstrating that the compounds dissociated into their monomeric forms under these conditions.

The ability of the squaraine dyes to generate singlet oxygen was assessed using the 1,3-diphenylisobenzofuran (DPBF) assay. In this method, squaraine solutions were incubated with DPBF and irradiated for different time intervals, followed by the monitoring of absorbance at 410 nm. A progressive decrease in DPBF absorbance was observed upon irradiation for all synthesized compounds, confirming their ability to produce this reactive oxygen species. However, it should be emphasized that the reference squaraine dye induced a sharp reduction in DPBF absorbance within a short irradiation period, with the decay being limited only by its intrinsic photoinstability.

**Figure 4:**
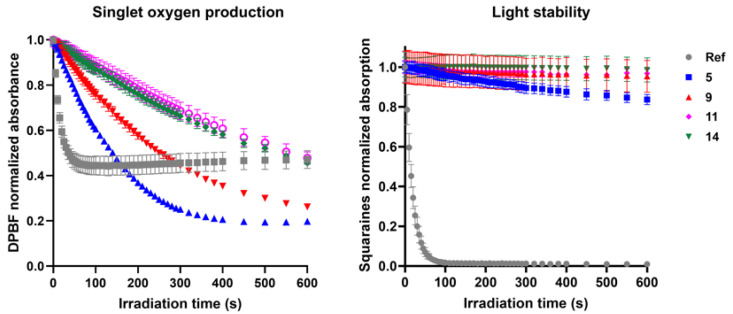
Singlet oxygen generation and light stability of *N*-propylbenzene indolenine-based squaraine dyes **5**, **9**, **11**, and **14** under LED irradiation in dimethylsulfoxide. Singlet oxygen production was evaluated by the decay of 1,3-diphenylisobenzofuran (DPBF) absorbance at 410 nm, and light stability was assessed by monitoring the absorbance of the dyes at their maximum absorption wavelength. A benzoselenazole-based squaraine dye was used as the reference (Ref).

Among the new derivatives, the non-substituted dye **5** and the amino-substituted analogue **9** displayed comparatively higher singlet oxygen generation (Figure 4). Nevertheless, when benchmarked against the reference (Φ_Δ (Ref)_ = 10%), all dyes synthesized in this work exhibited severely low quantum yields (Φ_Δ_ < 1%), highlighting their poor efficiency in photosensitization. On the other hand, all *N*-propylbenzene indolenine-based squaraines demonstrated good photostability, maintaining their structural integrity under irradiation.

### 2.3. Squaraine-Albumin Bonding Studies

To assess the interaction between the synthesized squaraine dyes and human serum albumin (HSA), two complementary approaches were employed. First, an in silico strategy was carried out using molecular docking to predict the interactions between the squaraines and the entire protein, with a specific grid defined for Sudlow’s site I and Sudlow’s site II, the main ligand-binding pockets that also accommodate several clinically relevant drugs. The second approach was performed in vitro through fluorometric measurements, in which the protein concentration was kept constant while the concentration of the potential probe was gradually increased, allowing for the collection of various binding and fluorescence-related parameters.

From the perspective of their use as fluorescent probes, an ideal outcome would be a strong and rapid interaction with albumin, as this would facilitate their detection and quantification. Conversely, for potential PDT applications, excessively strong binding could be detrimental, as it would limit the availability of free dye to interact with cellular targets and potentially reduce therapeutic efficacy, since the photosensitizer could remain sequestered in the bloodstream for prolonged periods.

In silico, regardless of the amine introduced into the central ring, all squaraine dyes exhibited lower binding energies (BE) to albumin compared with warfarin and ibuprofen, supporting the prediction of strong interactions with this protein (Table 2). Warfarin and ibuprofen were chosen as reference ligands since they are well-established standard probes for Sudlow’s sites I and II, respectively, on HSA [39]. Notably, the aminomethylpyrene-bearing squaraine **14** showed the strongest binding, with calculated BE ranging from −15.57 to −12.59 kcal/mol. In contrast, the introduction of amino and methylamino substituents (squaraines **9** and **11**, respectively), which produced comparable predictions, proved to be the least favorable modification, with binding energies between −12.64 and −9.75 kcal/mol, irrespective of the protein site considered. Interestingly, despite presenting higher binding energies than dye **14**, the unsubstituted squaraine **5** stood out when compared with the other amino-substituted derivatives, particularly at Sudlow’s site I, where a binding energy of −14.25 kcal/mol was predicted.

**Figure 5:**
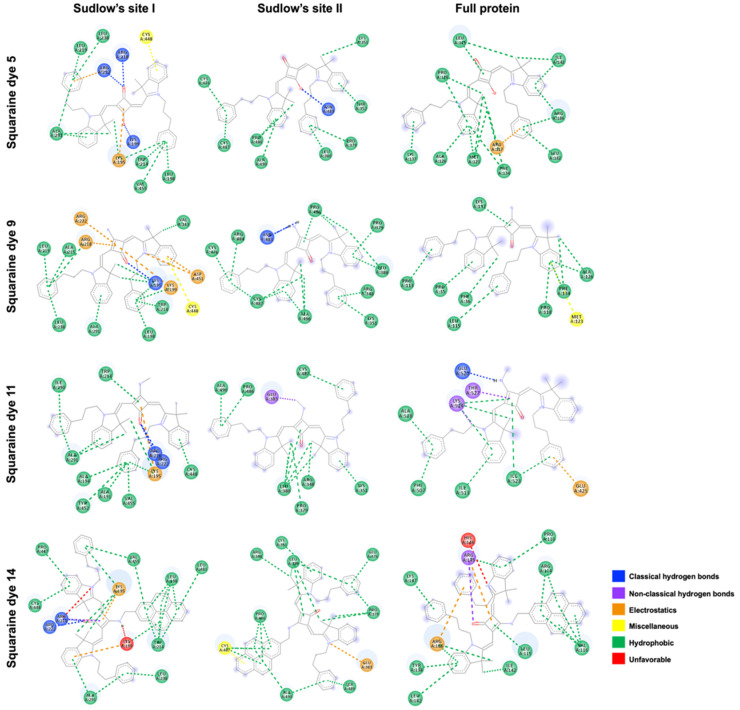
Two-dimensional interaction diagrams showing molecular docking results of *N*-propylbenzene indolenine-based squaraine dyes **5**, **9**, **11**, and **14** with human serum albumin (HSA) at Sudlow’s site I, Sudlow’s site II, and the full protein. Interactions are represented as classical hydrogen bonds, non-classical hydrogen bonds, electrostatics, miscellaneous, hydrophobic, and unfavorable contacts.

Concerning the nature of the interactions illustrated in Figure 5, hydrophobic contacts were generally predominant, regardless of the binding site. Interestingly, however, all dyes were predicted to establish hydrogen bonds within Sudlow’s site I, which explains the considerably lower binding energies observed at this site. Specifically, the carbonyl groups of the unsubstituted dye **5** and the methylamino and aminomethylpyrene derivatives **11** and **14** formed hydrogen bonds with residues ARG218 and ARG222, while the negatively charged oxygen atom of dye **5** interacted with LYS199. In addition to potential electrostatic interactions, noteworthy was the predicted engagement of the central ring’s conjugated double bond with residues LYS195 and LYS199. Moreover, π–sulfur interactions were expected between the conjugated aromatic rings of the heterocyclic moieties of squaraine dyes **5** and **9** and residue CYS448. Finally, within the same binding site, an unfavorable interaction was predicted between the amine hydrogen of dye **14** and LYS99 residue. Interestingly, this residue was also expected to engage in electrostatic interactions with the aromatic ring of one of the heterocyclic units, as well as in hydrophobic contacts with the pyrene moiety.

At Sudlow’s site II, classical hydrogen bonds were predicted between residue ASN483 and both the negatively charged oxygen atom of the unsubstituted dye **5** and the amino hydrogens of dye **9**. For dyes **11** and **14**, additional key interactions were anticipated, including non-conventional hydrogen bonds between the methyl group of the methylamino substituent and GLU383, an electrostatic interaction between the positively charged nitrogen of the heterocyclic unit and GLU383, and a π–sulfur interaction between the pyrene moiety and CYS487 residue, respectively.

At the third binding site, only the dye functionalized with a methylamino group was predicted to establish hydrogen bonding between its labile hydrogen atom and GLU520. Non-conventional hydrogen bonds were also expected between THR527 and the squaric ring double-bond carbons, and between LYS524 and the methylene group directly attached to the non-charged heterocyclic nitrogen of dye **11**. In addition, a hydrogen bond was predicted between the carbonyl group of the pyrene-containing dye and ARG145. Except for dye **9**, all dyes were expected to engage in electrostatic interactions, particularly involving the phenyl groups of the *N*-propylbenzyl substituents with residues ARG117, GLU425, and ARG186 for dyes **5**, **11**, and **14**, respectively. Notably, dye **9** was the only compound predicted to establish a π–sulfur interaction, involving the aromatic ring of its heterocyclic unit and residue MET123. Finally, an unfavorable interaction was predicted, arising from charge repulsion between residue HIS146 and the positively charged nitrogen atom of one of the heterocycles.

**Figure 6:**
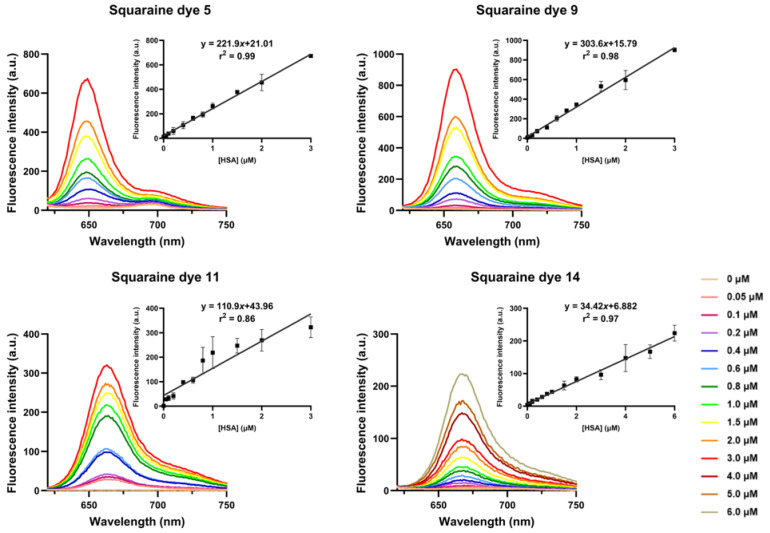
Fluorescence response of squaraine dyes **5**, **9**, **11**, and **14** upon titration with human serum albumin (HSA). The main panels show the emission spectra of each dye in the presence of increasing HSA concentrations (0–6 μM, as indicated by color scale). Insets display the corresponding binding curves, obtained by plotting fluorescence intensity at the emission maximum *versus* HSA concentration, demonstrating a dose-dependent increase in fluorescence upon protein binding. Error bars represent standard deviations from at least duplicate measurements.

Experimentally, all evaluated squaraine dyes exhibited very weak fluorescence in phosphate buffer alone (Figure 6). This behavior is primarily attributed to their strong tendency to aggregate in aqueous media, where π–π stacking and hydrophobic interactions typically lead to efficient fluorescence quenching and a reduction in molar absorptivity compared to organic solvents (Table 1 and Table 3). For the higher-wavelength methylamino and aminomethylpyrene dyes **11** and **14**, this quenching effect was so pronounced that their maximum emission wavelength could not be determined, with relative fluorescence quantum yields approaching zero. In contrast, dyes **5** and **9** were weakly fluorescent, displaying favorable Stokes shifts of 91 and 37 nm, respectively, a feature that could enhance their suitability as fluorescent probes by reducing self-absorption.

Upon binding to HSA, the most critical observation was an enhancement in fluorescence ability, confirming that the protein environment effectively monomerizes the aggregated dyes and restricts internal rotations, thereby suppressing non-radiative decay pathways (Figure 6). Aminosquaraines **9**, **11**, and **14** showed a “turn-on” effect, with relative fluorescence quantum yields increasing from approximately 0.00 to 0.07, 0.04, and 0.01, respectively (Table 3). Unsubstituted Squaraine dye **5** showed the largest relative increase, with the quantum yield increasing about tenfold to 0.20 in the bound state. Furthermore, the binding led to significant spectral changes: dye **5** underwent a substantial 47 nm blue shift in maximum emission wavelength and a 50% reduction in Stokes shift, consistent with the dye transitioning from an aggregated state to a restricted, less polar microenvironment within the protein.

Fluorescence intensity was measured at 0 min, 3 h, and 24 h to determine the incubation time required for maximum interaction between each dye and HSA. Overall, all squaraines demonstrated binding to the protein, as evidenced by the increase in fluorescence intensity with higher protein concentrations. For amino- and methylamino-bearing dyes **9** and **11**, maximum interaction occurred immediately upon addition to the protein solution, indicating that no incubation time is required and that these compounds bind to albumin almost instantaneously. In contrast, unsubstituted and aminomethylpyrene-containing dyes **5** and **14**, respectively, reached their maximum fluorescence only after 24 h of incubation at room temperature, protected from light.

The strong interaction between the synthesized squaraine dyes and the albumin observed in the fluorescence studies was quantitatively analyzed to assess their potential as analytical probes (Table 4). Upon complexation, all dyes exhibited a significant increase in fluorescence intensity, quantified by the ratio of the maximum intensity in the presence of protein (F) to the initial intensity (F_0_). Methylamino squaraine **11** displayed the largest relative enhancement, with an F/F_0_ ratio of about 361, a value consistent with its near-zero initial fluorescence being efficiently “turned on” by the protein binding. Dye **9** also showed remarkable performance, achieving the highest absolute fluorescence intensity (F = 917.53 a.u.) and an exceptional F/F_0_ ratio of near 99. The analytical performance confirmed the high sensitivity of these dyes, with amino-bearing dye **9** standing out as the most sensitive probe (S = 3.18 × 10^5^ nM^−1^), closely followed by the unsubstituted one **5** (2.09 × 10^5^ nM^−1^), values that translate into favorable detection abilities. The detection limits ranged from 266 nM for dye **5** to 420 nM for dye **11**, while the quantification limits spanned from 886 nM to 1401 nM (values corresponding to squaraines **5** and **11**, respectively). Finally, the calculated binding constants confirmed a strong dye–protein affinity consistent with the observed spectral changes, with values ranging from 10^5^ to 10^6^ M^−1^ and squaraine **9** showing the highest affinity at 6.23 × 10^5^ M^−1^. Collectively, the quantitative parameters, including the high F/F_0_ ratios, remarkable sensitivity, and strong binding affinities, highlight these squaraine compounds as highly effective turn-on fluorescent probes for the selective detection and quantification of this protein in an aqueous solution.

### 2.4. In Vitro Photodynamic Effects

For potential applicability as photosensitizers in PDT, these candidates are expected to display cytotoxic effects only after photoactivation, namely, and preferably restricted to target tissue cells, without inducing damage to healthy counterparts. To evaluate this, prostate adenocarcinoma PC-3 cells and non-tumoral NHDF fibroblasts were incubated with increasing concentrations of the squaraine dyes for 24 h. Subsequently, the cells were irradiated using an LED system centered at 663 nm, a wavelength close to the absorption maximum of the monomeric dye forms. Twenty-four hours post-irradiation, cellular metabolic activity was assessed by the MTT assay.

**Figure 7:**
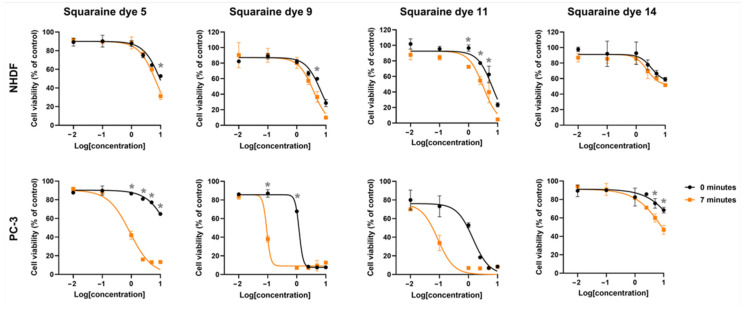
Sigmoidal dose–response curves of dark and light-induced cytotoxicity of *N*-propylbenzene indolenine-based squaraine dyes **5**, **9**, **11**, and **14** against normal human dermal fibroblasts (NHDF) and prostate adenocarcinoma cells (PC-3). Cell viability was assessed by the MTT assay after 24 h of incubation with increasing dye concentrations, followed by either no irradiation (black curves) or 7 min of LED irradiation (orange curves). Data are the mean cell viability (% of control) ± standard deviation from at least two independent experiments, expressed as a percentage relative to the untreated control cells. Asterisk indicates statistically significant differences between dark and light-treated samples at the same concentration (two-way ANOVA, *p*-value < 0.05).

Photodynamically, all dyes demonstrated light responsiveness to varying degrees, as evidenced by reduced cell viability following irradiation. The unsubstituted squaraine dye **5** showed marked differences in photocytotoxic activity between the two cell lines: while no reduction in viability greater than 30% was observed in normal fibroblasts up to the highest tested concentration of 2.5 μM, a much more pronounced effect was detected in PC-3 cells, where approximately 60% reduction in viability occurred at just 1.0 μM (Figure 7). This effect is reflected in the calculated photodynamic ratio, which indicates that this dye is at least 11.1-fold more cytotoxic upon irradiation compared to its inherent dark cytotoxicity (Table 5). Moreover, the tumor selectivity ratio revealed that dye **5** was about 8.2-fold more toxic toward tumor cells than toward normal fibroblasts under light treatment.

The introduction of different amine substituents into the four-membered central ring resulted in markedly distinct biological outcomes. Functionalization with amino and methylamino groups (squaraines **9** and **11**, respectively) rendered the dyes highly promising, showing 13- and 16-fold increases in cytotoxicity upon irradiation, as well as 40.8- and 36-fold higher photocytotoxicity toward PC-3 cells compared to normal fibroblasts. In contrast, functionalization of the squaric ring with an aminomethylpyrene group (dye **14**) did not prove to be as advantageous. Despite the slight differences in cell viability reduction observed for this latter dye across both cell lines, no significant effects were detected when compared to the other dyes evaluated. This was reflected in IC_50_ values exceeding 10 μM, the highest concentration tested.

## 3. Discussion

This study aimed to investigate how subtle structural modifications in *N*-propylbenzene indolenine-based squaraine dyes affect their interaction with HSA. Understanding these effects provides valuable insights not only into their potential use as fluorescent probes for the detection and quantification of this protein, but also into their possible transport and biodistribution mechanisms in the body when envisioned as drug candidates for PDT. Furthermore, the work included an evaluation of their in vitro photodynamic activity against prostate cancer cells.

Photophysically, the synthesized squaraine dyes exhibited several key properties required for applications in both areas of interest: unlike porphyrins, the main class of clinically approved photosensitizers that absorb at shorter wavelengths [40,41], they displayed maximum absorption within a spectral region where most biomolecules do not absorb efficiently, high molar absorptivity coefficients indicative of strong light-harvesting capability, and emission maxima moderately shifted from their absorption bands, depending on the solvent. Squaraines’ fluorescence quantum yields were also moderate and solvent-dependent, while they demonstrated remarkable photostability. The latter was indeed expected, as indolenine-based dyes are generally known for their excellent light stability [42,43], in contrast to other heterocyclic derivatives such as benzoselenazole and benzothiazole [44,45]. It is therefore understood that regarding this property, the heterocyclic core from which these dyes are derived plays a decisive role in ensuring such robustness.

The synthesized dyes were found to exhibit a marked tendency to aggregate in aqueous media, a common feature among squaraine compounds due to their highly planar and π-conjugated structures that promote strong intermolecular interactions. Such aggregation is generally disadvantageous for their intended applications, as it often results in fluorescence quenching through non-radiative deactivation of excited states [46,47] and may also reduce the generation of reactive oxygen species by limiting the availability of excited singlet and triplet states [48]. Nevertheless, for these particular structures, the impact of aggregation on their photobiological performance may not be critical, since high molar absorption coefficients were still obtained at the absorption maximum of the monomeric form even in buffer solution. Interestingly, and quite distinctly, methylamino squaraine dye **11** showed the lowest tendency to aggregate, displaying a well-defined absorption band corresponding to its monomeric form, in contrast to all other derivatives.

Despite the promising in silico interaction profile predicted for dye **14**, bearing the aminomethylpyrene group, this high potential was not fully corroborated by the in vitro results. Instead, methylamino-functionalized dye **11** exhibited markedly higher binding constants, indicating a stronger actual interaction with the protein than computationally anticipated. This discrepancy highlights the influence of factors beyond static docking predictions, such as solvation effects, dye aggregation, and the conformational flexibility of HSA, on the real binding behavior in solution. The pronounced interaction of this dye with the protein was further evidenced by an approximately 360-fold fluorescence enhancement, far exceeding that of the other dyes tested, none of which surpassed a 100-fold increase relative to their baseline emission. From the perspective of their potential as fluorescent probes, the unsubstituted dye **5** proved most effective in vitro, enabling the detection and quantification of albumin at considerably lower concentrations than its amino-substituted squaraine analogues, which displayed higher detection and quantification limits. However, a notable limitation was the slow kinetics of binding, as the interaction required at least one hour of incubation to reach equilibrium, a drawback that may restrict its suitability for diagnostic, biochemical, or biotechnological applications demanding rapid response times.

Far below the expectations, only low to moderate fluorescence quantum yields were determined after protein addition, significantly lower than those observed for other analogous dyes recently investigated by some of us. For comparison, squaraine derivatives functionalized on the central ring with dimethylbarbituric, dimethylthiobarbituric, and diphenylthiobarbituric acids, as reported by Gomes et al. [13,49], exhibited remarkable increases in fluorescence quantum yield, from about 1% up to 57% in the presence of albumin. These compounds also displayed binding constants one order of magnitude higher than those of the dyes reported herein (10^6^ M^−1^), except for dye **11**, whose binding affinity was comparable to that of the barbituric derivatives. In comparison with the *N*-hexyl chain indolenine- and benz[*e*]indole-based derivatives reported by Sousa et al. [50], although the squaraines presented herein exhibited greater fluorescence enhancement in the presence of protein relative to its absence, the previously reported analogues displayed properties more consistent with the requirements for probe applications. Indeed, those compounds showed considerably higher fluorescence quantum yields, greater sensitivity, and overall, lower detection and quantification limits.

From a photodynamic perspective, the dyes presented herein exhibited significant potential, except for the aminomethylpyrene-bearing derivative **14**, which showed no appreciable cytotoxicity toward the tumor cell line, regardless of irradiation conditions. Among the remaining compounds, the amino- and methylamino-substituted dyes, squaraines **9** and **11**, respectively, stood out, as they not only displayed pronounced phototoxicity but also exhibited a tendency toward selective activity in tumor cells compared with the normal cell line. Although these dyes showed relatively low fluorescence quantum yields, this is consistent with their strong photodynamic activity. After light absorption, excited dyes can either emit fluorescence or undergo intersystem crossing to the triplet state, generating ROS. Efficient intercrossing system competes with fluorescence, so dyes with lower fluorescence typically produce more ROS, explaining the inverse relationship between fluorescence efficiency and photodynamic cytotoxicity [51,52]. Although the two cell lines originate from different embryonic tissues, the lower cytotoxicity observed in NHDF suggests a preference for prostate adenocarcinoma cells. Although the two cell lines originate from different embryonic tissues, the markedly lower cytotoxicity observed in NHDF indicates a preferential accumulation and activity of these dyes in PC-3 cells. Such pronounced tumor selectivity underscores the potential safety of these dyes for the skin, an organ frequently exposed to light both during PDT and in daily life post-treatment. The introduction of amino substituents into the squaric ring, while enhancing certain molecular interactions, led to an undesirable increase in dark cytotoxicity. Importantly, this modification did not compromise photodynamic efficacy: upon irradiation, the dyes exhibited cumulative cytotoxic effects, maintaining their therapeutic potential. Conversely, the unsubstituted squaraine **5**, although less selective, emerged as particularly attractive for photodynamic applications due to its minimal dark toxicity across both cell lines, offering a favorable safety profile even if its light-activated potency was comparatively lower.

Regarding their photodynamic mechanism of action, further studies are clearly required to elucidate the underlying processes. Although the measured singlet oxygen quantum yields were relatively low, our experience suggests that this reactive oxygen species may still contribute to the biological activity of these photosensitizing candidates [45,53]. Since both type I and type II photodynamic reactions may occur, these molecules are particularly attractive from a clinical perspective for treating tumors with hypoxic microenvironments [54,55,56]. Type II mechanisms are oxygen-dependent, leading mainly to the formation of singlet oxygen, whereas type I processes involve electron or hydrogen transfer reactions that generate reactive oxygen species such as superoxide anion, hydroxyl radicals, or hydrogen peroxide, and can therefore proceed even under oxygen-limited conditions. Indeed, the intracellular site and nature of ROS generation are often more critical than the absolute amount produced [57]. In contrast to porphyrin-based photosensitizers, which mainly rely on singlet oxygen generation [58,59], squaraine dyes are predicted to mainly engage in type I photoreactions, potentially enhancing their performance under hypoxic conditions commonly found in tumors [60,61,62]. Therefore, additional investigations are needed to fully understand how these dyes exert their effects upon photodynamic activation. The intrinsically low cytotoxicity and favorable safety profile of these dyes toward healthy cells also highlight their potential for future exploration as photoantibacterial and -antifungal candidates within the broader scope of PDT applications.

Overall, the poor fluorescence performance of the aminomethylpyrene-functionalized dye, both in organic and aqueous environments, together with its weak photodynamic activity, does not support its potential for further applications in this context. Nevertheless, it is worth noting that a wide range of other dye molecules, such as porphyrins [63], imidazoles [64], quinolines [65], and naphthalenes [66] functionalized with pyrene moieties, have been reported in the literature as effective stabilizers or destabilizers of G-quadruplex structures, encouraging further exploration of this pyrene-functionalized dye for applications in nucleic acid targeting.

A few limitations of this study should be acknowledged. The in vitro assays provide useful preliminary insights but cannot fully capture the complexity of in vivo environments, including biodistribution, metabolism, and protein interactions. The low singlet oxygen quantum yields also indicate the need for further mechanistic studies to clarify type I *versus* type II pathways. Future work will address these gaps through more detailed in vitro experiments using specific ROS quenchers and scavengers, subcellular localization analysis, evaluation of cell death mechanisms, and assessment of cell cycle effects. Promising results will pave the way for studies in three-dimensional cell cultures and animal models, alongside structural optimization to enhance protein binding and overall phototherapeutic efficacy.

## 4. Materials and Methods

All reagents used in this work, except those synthesized during the experimental procedure, were commercially obtained and used without further purification. Dichloromethane employed in the organic synthesis reactions was first dried over anhydrous calcium chloride and subsequently refluxed over calcium hydride.

The progress of the synthetic reactions and the chromatographic assessment of the purity of the obtained crystals were monitored by thin-layer chromatography on aluminum plates coated with silica gel (Merck, 60 F_254_, thickness 0.25 mm; Merck, Darmstadt, Germany). Dichloromethane/methanol mixtures (5% and 2%) were used as mobile phases. After elution, the chromatograms were visualized under UV light at 254 and/or 366 nm. Plates containing compounds corresponding to quaternary ammonium salts were further developed with Dragendorff’s reagent [1:1 (*v*/*v*) mixture of 2% bismuth nitrate in 20% aqueous acetic acid and 40% aqueous potassium iodide], which produces an orange coloration.

Melting points (m.p.) were determined with a binocular microscope equipped with a heated stage and digital thermometer (URA Technic, Lisbon, Portugal).

UV–Vis spectra were recorded on a Perkin-Elmer Lambda 25 spectrophotometer (Perkin-Elmer, Waltham, MA, USA) using Hellma Analytics quartz cuvettes (1 cm path length; Hellma Analytics, Müllheim, Germany). Spectral data are reported as maximum absorption wavelength (λ_max_, nm) and molar absorptivity (ε, M^−1^·cm^−1^). The latter was calculated according to the Lambert–Beer law from calibration plots of absorbance at λ_max_ *versus* known dye concentrations.

Fluorescence spectra were acquired on a Varian Cary Eclipse fluorometer (Agilent Technologies, Santa Clara, CA, USA) using 1 cm path length, four-sided polished Hellma Analytics quartz cuvettes.

IR spectra were recorded on a Shimadzu IRAffinity-1S spectrophotometer (Shimadzu, Kyoto, Japan) using KBr pellets. Spectral data are reported as follows: sample form [KBr]; absorption maxima (ʋ_max_, cm^−1^); band intensity [strong (s), medium (m), weak (w)], and, when possible, characteristic functional group assignments.

^1^H and ^13^C NMR spectra were obtained on a Bruker Avance III 400 spectrometer (University of Beira Interior; Bruker, Billerica, MA, USA) using DMSO-*d*_6_ as the solvent. Chemical shifts (δ, ppm) are reported with reference to solvent signals (^1^H DMSO-*d*_6_ 2.50 ppm and ^13^C DMSO-*d*_6_ 39.52 ppm), along with the number of protons, multiplicity [singlet (s), broad singlet (br s), doublet (d), broad triplet (br t), triplet (t), quintet (qt), multiplet (m)], coupling constants (*J*, Hz), and assignments. ^13^C NMR data were supported by DEPT-135 experiments to distinguish CH_3_, CH_2_, and CH signals.

High-resolution mass spectra (HRMS) were obtained using a Bruker solariX XR spectrometer (Bruker, Billerica, MA, USA) at the Mass Spectrometry Services of C.A.C.T.I., University of Vigo, equipped with an electrospray ionization (ESI) and Fourier transform ion cyclotron resonance analyzer. Data are reported as *m*/*z* values of the molecular ion, molecular formula, and calculated exact mass.

## 5. Synthesis of *N*-Propylbenzene Indolenine-Based Aminosquaraines

### 5.1. 2,3,3-Trimethyl-1-(3-phenylpropyl)-indol-1-ium iodide (***3***)

In a 50 mL round bottom flask, 2,3,3-trimethylindolenine (**1**; 2.00 g; 12.6 mmol) was mixed with the alkylating agent 3-iodo-1-phenylpropane (**2**; 6.20 g; 25.2 mmol), in acetonitrile (25 mL), under stirring and reflux (90 °C), for 6 days. After this time, the reaction mixture was removed from heating and placed in an ice bath, and diethyl ether was added to promote precipitation. The resulting solid was filtered at reduced pressure and washed several times with diethyl ether, and then recrystallized by dissolving them in the minimum amount of methanol and adding diethyl ether until crystallization began. After vacuum filtration followed by drying under vacuum, cream-colored needle-shaped crystals of compound **3** were obtained, with a yield of 79%. M.p.: 135–137 °C; IR ʋ_max_ (KBr): 3024 (s, ArCH), 2970–2860 (m, CH), 1626 (m, C=C), 1593 (m, ArC=C), 1497 (m), 1479 (s), 1462 (s), 1366 (w), 1126 (w), 1042 (w), 937 (w), 768 (s), 750 (s), 698 (s), 496 (m) cm^−1^; ^1^H NMR (DMSO-*d*_6_, 400.13 MHz) δ: 7.98–7.93 (1H, m, ArH), 7.85–7.82 (1H, m, ArH), 7.65–7.61 (2H, m, ArH), 7.33–7.27 (4H, m, ArH), 7.24–7.20 (1H, m, ArH), 4.49 (2H, t, *J* = 7.8, NCH_2_CH_2_CH_2_Ph), 2.81 (2H, t, *J* = 8.0, NCH_2_CH_2_CH_2_Ph), 2.81 (3H, s, CH_3_), 2.17 (2H, qt, *J* = 8.0, NCH_2_CH_2_CH_2_Ph), 1.52 (6H, s, C(CH_3_)_2_) ppm. ^13^C NMR (DMSO-*d*_6_, 100.62 MHz) δ: 196.56, 141.83, 141.04, 140.51, 129.37 (ArCH), 128.90 (ArCH), 128.37 (ArCH), 128.23 (ArCH), 126.14 (ArCH), 123.48 (ArCH), 115.32 (ArCH), 54.14 (C(CH_3_)_2_), 47.30 (NCH_2_CH_2_CH_2_Ph), 31.77 (NCH_2_CH_2_CH_2_Ph), 28.70 (NCH_2_CH_2_CH_2_Ph), 21.96 (CH_3_), 13.98 (C(CH_3_)_2_) ppm; HRMS *m*/*z*: 278.190601 [M–I]^+^ (C_20_H_24_N calc. 278.190326).

### 5.2. 4-[3,3-Dimethyl-1-(3-phenylpropyl)-indol-1-ium-2-ylmethylene]-2-[3,3-dimethyl-1-(3-phenylpropyl)indolin-2-ylidenemethyl]-3-oxocyclobut-1-en-1-olate (***5***)

In a 100 mL round bottom flask, the indolenine derivative salt **3** (1.50 g; 3.70 mmol) was mixed with 3,4-dihydroxycyclobut-3-ene-1,2-dione (**4**; 0.21 g; 1.85 mmol) in 30 mL of a mixture of *n*-butanol and pyridine (10%), under stirring and reflux (140 °C), for 6 h. The reaction mixture was removed from heating and placed in an ice bath, and diethyl ether was added to the flask to initiate the first precipitation. Then, for recrystallization, the resulting solid was dissolved with methanol and some drops of heated dichloromethane, and after complete dissolution, diethyl ether was added, and the flask was left in an ice bath. Green metallic crystals were obtained of compound **5**, after filtration under reduced pressure, with a yield of 85%. M.p.: 234–235 °C. Vis λ_max_ (DMSO): 646 nm; ε = 292,993 M^−1^·cm^−1^. IR ʋ_max_ (KBr): 3055 (w, ArCH), 3024 (w, ArCH), 2956–2864 (w, CH), 1597 (s, ArC=C), 1576 (m, ArC=C), 1493 (s), 1450 (s), 1427 (s), 1398 (m), 1354 (m), 1283 (s), 1231 (m), 1219 (m), 1161 (s), 1098 (s), 1076 (s), 1059 (s), 1018 (m), 966 (m), 924 (m), 791 (m), 752 (m), 698 (m), 683 (m), 554 (w) cm^−1^. ^1^H NMR (DMSO-*d*_6_, 400.13 MHz) δ: 7.53 (2H, d, *J* = 7.2, ArH), 7.34 (2H, t, *J* = 7.6, ArH), 7.30–7.28 (4H, m, ArH), 7.25–7.19 (8H, m, ArH), 7.17 (2H, d, *J* = 7.6, ArH), 5.83 (2H, s, CH=C), 4.14 (4H, br t, NCH_2_CH_2_CH_2_Ph), 2.74 (4H, t, *J* = 8.0, NCH_2_CH_2_CH_2_Ph), 2.02 (4H, qt, *J* = 6.4, NCH_2_CH_2_CH_2_Ph), 1.69 (12 H, s, C(CH_3_)_2_) ppm. ^13^C NMR (DMSO-*d*_6_, 100.62 MHz) δ: 180.68, 178.70, 169.10, 142.19, 141.44, 140.93, 128.46 (ArCH), 128.17 (ArCH), 127.96 (ArCH), 126.04 (ArCH), 123.70 (ArCH), 122.29 (ArCH), 110.19 (ArCH), 86.15 (CH=C), 48.74 (C(CH_3_)_2_), 42.71 (NCH_2_CH_2_CH_2_Ph), 32.29 (NCH_2_CH_2_CH_2_Ph), 28.32 (NCH_2_CH_2_CH_2_Ph), 26.54 (C(CH_3_)_2_) ppm. HRMS *m*/*z*: 632.3399654 [M]^+^ (C_44_H_44_N_2_O_2_ calc. 632.339730).

### 5.3. 2-{3-[3,3-Dimethyl-1-(3-phenylpropyl)indolin-2-ylidenemethyl]-2-methoxy-4-oxocyclobut-2-en-1-ylidenemethyl}-3,3-dimethyl-1-(3-phenylpropyl)-indol-1-ium trifluoromethanesulfonate (***7***)

In a 100 mL round bottom flask, compound **5** (0.50 g; 0.79 mmol) was mixed with methyl trifluoromethanesulfonate (**6**; 0.39 g; 2.37 mmol) under stirring in 30 mL of dry dichloromethane. The reaction took place at room temperature, under a nitrogen atmosphere, for 3 h. Once finished, the reaction mixture was treated, placing it in an extraction ampoule and washed with an ice-cold 5% (*w*/*v*) sodium hydrogen carbonate solution (100 mL). Then, distilled water was used to wash the organic phase that was posteriorly dried with anhydrous sodium sulfate. The solvent was removed to dryness using a rotary evaporator, yielding a golden film, which was converted into a paste upon the addition of ether. To precipitate this paste, a mixture of dichloromethane and methanol, followed by petroleum ether, was added. The solid was then washed several times with heated diethyl ether to remove impurities and subsequently recrystallized from a mixture of dichloromethane and diethyl ether. After filtration to reduced pressure, compound **7** was obtained as dark green metallic crystals, with a yield of 75%. M.p.: 151–152 °C. IR ʋ_max_ (KBr): 3057 (w, ArCH), 3024 (w, ArCH), 2968–2859 (w, CH), 1757 (m, C=O), 1647 (m, ArC=C), 1601 (m, ArC=C), 1524 (s), 1499 (s), 1360 (m), 1314 (m, C–O), 1296 (m, C–O), 1148 (m), 1049 (m), 922 (m), 824 (m), 791 (m), 748 (m), 691 (m), 637 (s), 554 (w) cm^−1^. ^1^H NMR (DMSO-*d*_6_, 400.13 MHz) δ: 7.62 (2H, d, *J* = 7.2, ArH), 7.49 (2H, t, *J* = 8.2, ArH), 7.45 (2H, t, *J* = 7.0, ArH), 7.35–7.29 (6H, m, ArH), 7.24–7.22 (6H, m, ArH), 5.80 (2H, s, CH=C), 4.55 (3H, s, OCH_3_), 4.25 (4H, t, *J* = 7.8, NCH_2_CH_2_CH_2_Ph), 2.73 (4H, t, *J* = 7.8, NCH_2_CH_2_CH_2_Ph), 2.04 (4H, qt, *J* = 7.6, NCH_2_CH_2_CH_2_Ph), 1.65 (12 H, s, C(CH_3_)_2_) ppm. ^13^C NMR (DMSO-*d*_6_, 100.62 MHz) δ: 179.09, 176.91, 173.13, 160.71, 141.92, 141.39, 140.65, 128.43 (ArCH), 128.36 (ArCH), 128.22 (ArCH), 126.09 (ArCH), 125.56 (ArCH), 122.41 (ArCH), 111.85 (ArCH), 86.75 (CH=C), 61.03 (OCH_3_), 49.62 (C(CH_3_)_2_), 43.65 (NCH_2_CH_2_CH_2_Ph), 32.01 (NCH_2_CH_2_CH_2_Ph), 28.41 (NCH_2_CH_2_CH_2_Ph), 25.52 (C(CH_3_)_2_) ppm. HRMS *m*/*z*: 647.363165 [M–CF_3_SO_3_]^+^ (C_45_H_47_N_2_O_2_ calc. 647.363205).

### 5.4. 2-{2-Amino-3-[3,3-dimethyl-1-(3-phenylpropyl)indolin-2-ylidenemethyl]-4-oxocyclobut-2-en-1-ylidenemethyl}-3,3-dimethyl-1-(3-phenylpropyl)-indol-1-ium Iodide (***9***)

In a 100 mL round bottom flask, the *O*-methylated derivative **7** (0.20 g; 0.25 mmol) was mixed with 2M ammonia solution in methanol (**8**; 0.013 g; 0.75 mmol), in dry dichloromethane (30 mL), under stirring, at room temperature, under a nitrogen atmosphere, for 3 h. The content of the flask was transferred to an extraction ampoule and washed three times with distilled water; the collected organic phase was dried over anhydrous sodium sulfate, filtered, and the solvent removed to dryness using a rotary evaporator. To the resulting residue, 10 mL of methanol and 10 mL of a 14% potassium iodide aqueous solution were added (1.4 g; 8.43 mmol), and the mixture was stirred at room temperature for 30 min. After decanting, the resulting residue, which was shown chromatographically to be a complex mixture of several compounds, was washed with distilled water and crushed with diethyl ether. After decanting, the solid was treated with heated ether and a minimal amount of dichloromethane, then placed in an ice bath until precipitation began. After vacuum filtration followed by drying under vacuum, pure bluish/greenish crystals were obtained, with a yield of 43%. M.p.: 156–157 °C. Vis λ_max_ (DMSO): 652 nm; ε = 232,117 M^−1^·cm^−1^. IR ʋ_max_ (KBr): 3256 (w, NH), 3102 (m, ArCH), 3024 (w, ArCH), 2961–2860 (w, CH), 1641 (s, ArC=C), 1535 (s, ArC=C), 1497 (s), 1454 (s), 1366 (s), 1314 (s), 1283 (s), 1159 (s), 1121 (w), 1098 (w), 1063 (s), 964 (m), 924 (w), 839 (w), 793 (m), 746 (m), 687 (w) cm^−1^. ^1^H NMR (DMSO-*d*_6_, 400.13 MHz) δ: 9.07 (2H, s, NH_2_, exchange with D_2_O), 7.59 (2H, d, *J* = 7.6, ArH), 7.41 (2H, dt, *J* = 7.6, 1.2, ArH), 7.35 (2H, d, *J* = 7.6, ArH), 7.31–7.25 (6H, m, ArH), 7.22–7.17 (6H, m, ArH), 6.06 (2H, s, CH=C), 4.28 (4H, t, *J* = 7.2, NCH_2_CH_2_CH_2_Ph), 2.74 (4H, t, *J* = 8.2, NCH_2_CH_2_CH_2_Ph), 2.05 (4H, qt, *J* = 7.7, NCH_2_CH_2_CH_2_Ph), 1.70 (12 H, s, C(CH_3_)_2_) ppm. ^13^C NMR (DMSO-*d*_6_, 100.62 MHz) δ: 172.99, 171.94, 170.91, 159.45, 141.84, 141.63, 140.93, 128.38 (ArCH), 128.17 (ArCH), 128.12 (ArCH), 126.01 (ArCH), 124.82 (ArCH), 122.34 (ArCH), 111.15 (ArCH), 87.77 (CH=C), 49.21 (C(CH_3_)_2_), 43.27 (NCH_2_CH_2_CH_2_Ph), 32.11 (NCH_2_CH_2_CH_2_Ph), 28.75 (NCH_2_CH_2_CH_2_Ph), 25.99 (C(CH_3_)_2_) ppm. HRMS *m*/*z*: 632.363460 [M–I]^+^ (C_44_H_46_N_3_O calc. 632.363540).

### 5.5. 2-{3-[3,3-Dimethyl-1-(3-phenylpropyl)indolin-2-ylidenemethyl]-2-(methylamino)-4-oxocyclobut-2-en-1-ylidenemethyl}-3,3-dimethyl-1-(3-phenylpropyl)-indol-1-ium Iodide (***11***)

In a 100 mL round bottom flask, the *O*-methylated derivative **7** (0.20 g; 0.25 mmol) was mixed with a 2 M solution of methylamine (**10**; 0.023 g; 0.75 mmol) in methanol, in dry dichloromethane (30 mL), at room temperature under stirring and nitrogen atmosphere, for 2 h. The mixture was washed twice with distilled water, recovering the organic phase and drying it with anhydrous sodium sulfate. After gravimetric filtration, the solvent was removed to dryness on the rotary evaporator, and the forming thick reddish paste was dissolved in 10 mL of methanol, and then 10 mL of ice-cold 14% potassium iodide solution (1.4 g; 8.43 mmol) was slowly added. The mixture was stirred for 2 h at room temperature. At the end of this time, the reaction mixture was transferred to an extraction ampoule, diluted in dichloromethane, and washed three times with distilled water. The organic phase was separated from the aqueous one, dried with anhydrous sodium sulfate, and the solvent was removed under reduced pressure. Successive recrystallizations were carried out with different solvents (acetonitrile/diethyl ether, methanol/petroleum ether/diethyl ether), and the last one with a mixture of dichloromethane and diethyl ether guaranteed the formation of pure *bordeaux* crystals with a 22% yield. M.p. 145–148 °C. Vis λ_max_ (DMSO): 663 nm; ε = 212,228 M^−1^·cm^−1^. IR ʋ_max_ (KBr): 3146 (w, NH), 3102 (w, ArCH), 3049 (w, ArCH), 2959–2859 (w, CH), 1632 (s, ArC=C), 1611 (m), 1568 (s), 1493 (s), 1479 (s), 1456 (s), 1398 (m), 1356 (m), 1285 (s); 1204 (w), 1159 (m), 1107 (m); 1080 (m); 1020 (m), 961 (m), 922 (m), 793 (m), 748 (m); 698 (m), 677 (m), 617 (w), 552 (w), 494 (w); 446 (w) cm^−1^; ^1^H NMR (DMSO-*d*_6_, 400.13 MHz) δ: 9.06 (1H, br s, NH, exchange with D_2_O), 7.61–7.55 (2H, m, ArH), 7.44–7.40 (4H, m, ArH), 7.32–7.26 (6H, m, ArH), 7.23–7.19 (6H, m, ArH), 6.00 (1H, s, CH=C), 5.95 (1H, s, CH=C), 4.30 (2H, t, *J* = 7.2, NCH_2_CH_2_CH_2_Ph), 4.20 (2H, t, *J* = 7.6, NCH_2_CH_2_CH_2_Ph), 3.28 (3H, s, NHCH_3_, observable after D_2_O exchange), 2.72 (4H, br t, *J* = 7.4, NCH_2_CH_2_CH_2_Ph), 2.04–1.98 (4H, m, NCH_2_CH_2_CH_2_Ph), 1.69 (12 H, s, C(CH_3_)_2_) ppm. ^13^C NMR (DMSO-*d*_6_, 100.62 MHz) δ: 172.77, 172.73, 170.95, 168.50, 159.60, 158.67, 142.08, 141.69, 141.49, 140.88, 140.78, 128.42 (ArCH), 128.27 (ArCH), 128.14 (ArCH), 126.09 (ArCH), 125.23 (ArCH), 124.54 (ArCH), 122.42 (ArCH), 122.30 (ArCH), 111.51 (ArCH), 111.00 (ArCH), 88.62 (CH=C), 87.73 (CH=C), 49.52 (C(CH_3_)_2_), 49.10 (C(CH_3_)_2_), 43.47 (NCH_2_CH_2_CH_2_Ph), 43.34 (NCH_2_CH_2_CH_2_Ph), 32.25 (NCH_2_CH_2_CH_2_Ph), 32.08 (NCH_2_CH_2_CH_2_Ph), 30.46 (NHCH_3_), 28.86 (NCH_2_CH_2_CH_2_Ph), 28.37 (NCH_2_CH_2_CH_2_Ph), 26.18 (C(CH_3_)_2_), 25.76 (C(CH_3_)_2_) ppm. HRMS *m*/*z*: 646.378971 [M–I]^+^ (C_45_H_48_N_3_O calc. 646.379190).

### 5.6. Synthesis of 2-({3-[3,3-dimethyl-1-(3-phenylpropyl)indolin-2-ylidenemethyl]-4-oxo-2-[(pyren-1-ylmethyl)amino]cyclobut-2-en-1-ylidene}methyl)-3,3-dimethyl-1-(3-phenylpropyl)-indol-1-ium Iodide (***14***)

In a 100 mL round bottom flask, pyrenemethylamine hydrochloride (0.20 g; 0.75 mmol), 0.061 mL of dry pyridine (0.059 g; 0.75 mmol), and 6 mL of dry dimethylformamide were mixed at room temperature, under stirring and a nitrogen atmosphere. After complete dissolution, a solution of the *O*-methylated dye **5** (0.20 g; 0.25 mmol) in dry dichloromethane (25 mL) was slowly added and allowed to react for 3 h. The mixture was transferred to a 100 mL extraction ampoule, and dichloromethane was added to dilute, and 3 washes with distilled water were performed. The organic phase was recovered, dried with anhydrous sodium sulfate, filtered gravimetrically, and the solvent removed to dryness on a rotary evaporator. After the addition of 10 mL of methanol and 10 mL of a 14% potassium iodide aqueous solution (1.4 g; 8.43 mmol), the mixture was allowed to react, under stirring, for 30 min. A paste was obtained, which was dissolved with dichloromethane and washed with distilled water 3 times in an extraction ampoule. After separation of the organic phase, dried with anhydrous sodium sulfate, gravimetrically filtered, and the solvent removed in the rotary evaporator, the obtained residue was washed several times with a 1:1 mixture of ice-cold ether and methanol, and proceeding with recrystallizations with these same solvents, it was possible to obtain pure green crystals with a 37% yield. M.p.: 231–232 °C. Vis λ_max_ (DMSO): 661 nm; ε = 196,583 M^−1^·cm^−1^. IR ʋ_max_ (KBr): 3088 (w, NH), 3036 (w, ArCH), 2957 (w, CH), 2922 (w, CH), 1630 (m, ArC=C), 1611 (w), 1560 (m), 1491 (s), 1479 (s), 1458 (s), 1356 (w), 1281 (s), 1240 (m), 1150 (s), 1117 (m), 1096 (m), 1057 (w), 1020 (w), 968 (w), 924 (w), 843 (w), 795 (w), 746 (m), 683 (w), 552 (w) cm^−1^. ^1^H NMR (DMSO-*d*_6_, 400.13 MHz) δ: 9.84 (1H, br t, *J* = 5.6, NHCH_2_, exchange with D_2_O), 8.57 (1H, d, *J* = 9.2, ArH), 8.41–8.36 (4H, m, ArH), 8.21–8.12 (4H, m, ArH), 7.65 (1H, d, *J* = 7.6, ArH), 7.48–7.43 (3H, m, ArH), 7.34 (1H, t, *J* = 7.6), 7.30–7.24 (2H, m, ArH), 7.23–7.19 (4H, m, ArH), 7.14 (1H, t, *J* = 6.8), 7.02–6.99 (4H, m, ArH), 6.44–6.42 (2H, m, ArH), 6.32 (1H, s, CH=C), 5.88 (2H, d, *J* = 6.0, NHCH_2_*Pyrene*, collapse to s with D_2_O), 5.61 (1H, s, CH=C), 4.37 (2H, t, *J* = 6.8, NCH_2_CH_2_CH_2_Ph), 3.40 (2H, d, *J* = 6.8, NCH_2_CH_2_CH_2_Ph), 2.74 (2H, t, *J* = 8.2, NCH_2_CH_2_CH_2_Ph), 2.10 (2H, qt, *J* = 7.6, NCH_2_CH_2_CH_2_Ph), 1.76 (6H, s, C(CH_3_)_2_), 1.67 (2H, t, *J* = 8.4, NCH_2_CH_2_CH_2_Ph), 1.57 (6H, s, C(CH_3_)_2_), 0.90 (2H, qt, *J* = 7.2, NCH_2_CH_2_CH_2_Ph) ppm. ^13^C NMR (DMSO-*d*_6_, 100.62 MHz) δ: 173.42, 173.28, 170.73, 168.85, 160.03, 158.06, 142.27, 141.60, 141.46, 141.38, 140.86, 139.93, 130.91, 130.54, 130.34, 128.41 (ArCH), 128.30 (ArCH), 128.14 (ArCH), 128.00 (ArCH), 127.50 (ArCH), 127.33 (ArCH), 126.60 (ArCH), 125.77 (ArCH), 125.72 (ArCH), 125.61 (ArCH), 125.46 (ArCH), 125.26 (ArCH), 124.38 (ArCH), 124.29 (ArCH), 124.18 (ArCH), 123.81 (ArCH), 122.47 (ArCH), 122.16 (ArCH), 111.74 (ArCH), 110.76 (ArCH), 88.57 (CH=C), 88.29 (CH=C), 49.75 (C(CH_3_)_2_), 48.97 (C(CH_3_)_2_), 45.41 (NHCH_2_*Pyrene*), 43.61 (NCH_2_CH_2_CH_2_Ph), 42.48 (NCH_2_CH_2_CH_2_Ph), 32.11 (NCH_2_CH_2_CH_2_Ph), 31.13 (NCH_2_CH_2_CH_2_Ph), 28.94 (NCH_2_CH_2_CH_2_Ph), 27.35 (NCH_2_CH_2_CH_2_Ph), 26.11 (C(CH_3_)_2_), 25.77 (C(CH_3_)_2_) ppm. HRMS *m*/*z*: 846.441816 [M–I]^+^ (C_61_H_56_N_3_O calc. 846.441790).

## 6. Photochemical and Photophysical Analysis

Ultraviolet–visible absorption spectra were obtained at room temperature using a PerkinElmer Lambda 25 spectrophotometer, while fluorescence emission data were collected on a Horiba Jobin Yvon FluoroMax-4 fluorometer. Singlet oxygen and photostability tests and measurements were carried out on a BioRad xMark microplate reader (Bio-Rad, Hercules, CA, USA).

Analytical-grade reagents, including 1,3-diphenylisobenzofuran, Triton X-100, and zinc phthalocyanine, were purchased from commercial suppliers without further purification. Phosphate buffer (0.05 M, pH 7.3) was prepared by dissolving disodium hydrogen phosphate heptahydrate (Na_2_HPO_4_·7H_2_O; 4.540 g) and sodium dihydrogen phosphate (NaH_2_PO_4_; 1.130 g) in Millipore water (1.0 L) (Millipore, Burlington, MA, USA).

Squaraine dyes were dissolved in dimethyl sulfoxide, whereas zinc phthalocyanine required dimethylformamide as a solvent. All stock solutions were prepared at 0.67 mM concentration and stored in the dark at 4 °C until further use. For spectroscopic characterization, 1.0 µM stock solutions of the squaraine dyes in dimethyl sulfoxide were serially diluted into a series of solvents (chloroform, dimethyl sulfoxide, methanol, and phosphate buffer) to obtain at least five defined concentrations. The absorption spectra of these dilutions were recorded and used to calculate molar extinction coefficients according to the Beer–Lambert law. Additional working solutions at a 1.0 µM concentration were prepared in dimethyl sulfoxide and buffer, and 1.0% Triton X-100 was added where required to investigate dye aggregation.

Singlet oxygen production was assessed by monitoring the bleaching of 1,3-diphenylisobenzofuran (DPBF) at 410 nm. Fresh solutions of squaraine dyes (20 µM) and DPBF (0.1 mM) were introduced into 96-well plates, and decreases in absorbance were recorded before and after irradiation at defined time intervals. Quantum yields of singlet oxygen generation (Φ_Δ_) were determined by comparing the slopes of DPBF degradation for the squaraine dyes [S(dye)] with those of a benzoselenazole squaraine reference dye [S(ref), Φ_Δ_ = 0.10], according to established linear regression procedures (Equation (1)). In parallel, photodegradation of the squaraines was monitored by following changes in their absorbance over time, with normalization applied to enable direct comparison across samples.

(1) $$\Phi_{\Delta \left(dye\right)}=\Phi_{\Delta \left(ref\right)}\;\times \;\frac{S\left(dye\right)}{S\left(ref\right)}$$

Fluorescence quantum yields (Φ_F_) were determined from the integrated emission spectra recorded in chloroform, dimethylformamide, and dimethyl sulfoxide. Zinc phthalocyanine (Φ_F_ = 0.17 in dimethylformamide) was employed as the reference standard. The emission areas were integrated, and relative quantum yields were calculated following established methodologies, considering both absorbance at the excitation wavelength and the refractive index of the solvent. The calculation was performed using Equation (2), where “dye” and “ref” denote the squaraine dyes and the reference compound, respectively; *I* corresponds to the integrated emission intensity, *A* to the absorbance value at the excitation wavelength, and *η* to the refractive index of the solvent:

(2) $$\Phi_{F(\mathrm{dye})}=\;\Phi_{F(\mathrm{Std})}\left(\frac{I_{\mathrm{dye}}}{I_{\mathrm{ref}}}\right)\;\left(\frac{A_{\mathrm{ref}}}{A_{\mathrm{dye}}}\right)\;\left(\frac{\eta_{\mathrm{dye}}^{2}}{\eta_{\mathrm{ref}}^{2}}\right)$$

## 7. In Silico

Molecular docking of squaraine dyes with HSA was performed using AutoDock Tools 1.5.6 at Sudlow sites I and II, as well as across the whole protein. HSA crystal structures (PDB: 2BXD and 2BXG), co-crystallized with *R*-(+)-warfarin and *S*-(+)-ibuprofen, respectively, were used to validate docking parameters against the literature data. Protein preparation (removal of chain B and water molecules) was carried out in UCSF Chimera 1.13.1, while dye structures were generated in ChemDraw/Chem3D 20.1.1.125, minimized, and converted to PDBQT format with OpenBabel 3.1.1. AutoDock protocols included the addition of hydrogens, Gasteiger charge assignment, and definition of torsional flexibility. Grid boxes were centered at the ligand sites or whole protein, and docking runs employed a Lamarckian genetic algorithm (60 runs, population size of 150, a maximum of 27,000 generations, and a limit of 5,000,000 energy evaluations). The lowest BE conformations for each dye and reference ligand were selected for subsequent analysis. Further methodological details can be found in our recent publications [45,50].

## 8. Dye-Protein Fluorescence-Based Studies

For fluorometric experiments, the squaraine stock solutions were initially prepared in dimethylformamide and subsequently diluted with phosphate buffer for the protein–dye interaction analysis. Human serum albumin stock solution was then added to these mixtures, while keeping the dye concentration fixed at 2.0 µM and varying the protein concentration from 0 to 3.0 µM (0.0 to 3.0 µM for squaraines **5**, **9**, and **11**, and from 0 to 6.0 µM for squaraine **13**). Fluorescence intensities of the resulting solutions were recorded after 0, 3, and 24 h of incubation with the protein. The excitation wavelengths and excitation/emission slit widths used for the measurements were optimized for each dye, as listed in Table 6.

The squaraine-albumin binding constants was calculated using the Benesi–Hildebrand equation (Equation (3)) based on the data obtained from the fluorescence experiments:

(3) $$\frac{1}{F_{x}-F_{0}}=\frac{1}{F_{\infty }-F_{0}}+\frac{1}{K_{b}(F_{\infty }-F_{0})}\frac{1}{\left[HSA\right]}$$

where F_0_ is the fluorescence intensity of the squaraine in albumin-free buffer, F_x_ is the fluorescence observed after protein incubation at a specific concentration, F_∞_ is the fluorescence at the point of complete interaction, K_b_ is the binding constant, and [HSA] is the albumin concentration.

Quantification and detection limits (QL and DL, respectively) were determined from a calibration curve of maximum fluorescence intensity against protein concentration. The slope of this curve was then used to calculate the method’s sensitivity using Equations (4) and (5):

(4) $$QL=\frac{10\sigma }{S}$$

(5) $$DL=\frac{3\sigma }{S}$$

where σ refers to the blank standard deviation and S (sensitivity) to the linear regression slope between the fluorescence intensity and albumin concentration.

## 9. Squaraine Cellular Photocytotoxicity Evaluation

### 9.1. Preparation of Squaraine Solutions

All squaraine dyes were initially dissolved in DMSO to a concentration of 1.0 mM and stored at 4–8 °C. Working solutions were then freshly prepared by diluting the stock solutions in complete culture medium immediately prior to each experiment.

### 9.2. Cell Culture

Normal human dermal fibroblasts and PC-3 cells were maintained in 75 cm^2^ culture flasks at 37 °C in a humidified incubator with 5% carbon dioxide. The non-tumor cells were cultured in RPMI 1640 medium supplemented with 10% fetal bovine serum (FBS), 2 mM L-glutamine, 10 mM HEPES, 1 mM sodium pyruvate, and 1% antibiotic/antimycotic solution (10,000 U/mL penicillin G, 100 µg/mL streptomycin, and 25 µg/mL amphotericin B). Prostate adenocarcinoma cells were cultured in RPMI 1640 supplemented with 10% FBS and 1% Sp antibiotic. For all cell lines, the culture medium was refreshed every two days, and cells were subcultured before reaching confluence.

### 9.3. Cell Viability Assessment

The *in cell* photocytotoxicity of the squaraine dyes was assessed using the MTT assay. When cells reached 90–95% confluence, they were trypsinized using a trypsin-EDTA solution (0.125 g/L trypsin, 0.02 g/L EDTA), counted *via* trypan-blue exclusion in a Neubauer chamber, and diluted to 2 × 10^4^ cells/mL. A 100 µL aliquot of this suspension was seeded into each well of 96-well plates and incubated for 48 h to allow for cell attachment and resumption of proliferation.

After this period, the medium was removed, and cells were treated with various concentrations of the squaraine dyes (0.01, 0.1, 1, 2.5, 5, and 10 µM) in the appropriate culture medium. The maximum DMSO concentration used was 1.0%, which by itself does not induce cytotoxicity. Negative controls consisted of cells exposed only to culture medium. All experiments were performed in quadruplicate and independently repeated.

After 24 h of exposure to the dyes, the cells were subjected to two different irradiation conditions: the first group was not irradiated and was protected from ambient light for 21 min, and the second group was irradiated for 7 min.

Twenty-four hours post-irradiation, the culture medium was removed, cells were washed with 100 µL PBS, and 100 µL of MTT solution (5 mg/mL in serum- and antibiotic-free medium) was added per well. Cells were incubated for 4 h at 37 °C, after which the supernatant was discarded, and the formazan crystals were solubilized in dimethyl sulfoxide. Absorbance was measured at 570 nm using a Bio-Rad xMark microplate reader.

Cell viability was expressed as the percentage of absorbance in treated samples relative to the untreated negative control. Data were statistically analyzed by ordinary two-way ANOVA followed by the Šidák multiple comparisons test, comparing irradiated and non-irradiated conditions at the same concentration, with differences considered statistically significant at a *p*-value < 0.05. Half-maximal inhibitory concentration values reported in this work were obtained by fitting the data to a sigmoidal model with a 95% confidence interval. All assays were performed in quadruplicate and replicated independently at least twice. Phototoxicity and tumor selectivity ratios were quantified according to Equations (6) and (7):

(6) $$PTR\;=\;\frac{{IC}_{50}\;dark\;condition}{{IC}_{50}\;irradiated\;condition}$$

(7) $$TSR\;=\;\frac{{IC}_{50}\;for\;NHDF\;cells\;under\;irradiated\;condition}{{IC}_{50}\;for\;cancer\;cell\;line\;under\;irradiated\;condition}$$

#### Light Source

Irradiation was performed using a light-emitting diode array centered at 663 ± 8 nm to match the absorption maximum of the dyes. The LED (Roithner; 8.3 mW radiant flux) was integrated into flat-bottom 96-well plates and powered by a regulated current source. The irradiance was 15.8 mW/cm^2^, and the total energy delivered per well was 3.49 J after 7 min, ensuring precise light dosing for all experiments.

## 10. Conclusions

A novel series of squaraine dyes was synthesized and characterized, featuring two key structural modifications aimed at enhancing their interaction with biological targets: *N*-propylbenzyl chains were introduced systematically across the entire series, while an aminomethylpyrene unit was incorporated into the four-membered central ring of one derivative. Both modifications were conceived with the shared objective of increasing the aromatic character of the dyes, thereby promoting π–π stacking and electrostatic interactions with the multiple hydrophobic and aromatic binding pockets of HSA. Computational studies supported this rationale, particularly for the aminomethylpyrene-containing dye **14**, which exhibited a predicted decrease in binding energy values, indicative of more favorable interactions. However, these computationally anticipated effects were not fully reflected in the in vitro results, as the dyes exhibited only moderate binding affinity toward HSA, weak fluorescence emission, and higher detection and quantification limits than other squaraine analogues, ultimately falling short of the initial expectations.

Interestingly, the moderate binding affinity observed for these dyes may actually represent a favorable pharmacological feature in the context of PDT, as photosensitizers with transient, reversible interactions with serum proteins typically exhibit shorter circulation half-lives and more efficient clearance, reducing off-target accumulation while still allowing albumin to serve as an endogenous carrier and facilitate passive tumor targeting. From a functional perspective, all derivatives except the pyrene-containing dye demonstrated robust photodynamic activity, inducing pronounced cytotoxic effects selectively upon light activation and exhibiting preferential activity toward prostate cancer cells. These findings highlight the potential of these new squaraine derivatives as tumor-selective photosensitizers and warrant further investigation to elucidate their mechanism of action and optimize their phototherapeutic performance.

## Figures and Tables

**Table 1:** Photophysical properties of *N*-propylbenzene indolenine-based squaraine dyes **5**, **9**, **11**, and **14** in different solvents [dimethyl sulfoxide (DMSO), chloroform (CFM), and methanol (MeOH)]. Reported parameters include the absorption maximum (λ_abs_), emission maximum (λ_em_), Stokes shift (ΔS, nm), molar absorption coefficient (ε, ×10^5^ M^−1^·cm^−1^), fluorescence quantum yield (Φ_F_), and singlet oxygen quantum yield (Φ_Δ_).

		Squaraine Dyes			
Solvent		5	9	11	14
DMSO	λ_abs_	646	652	663	661
	λ_em_	659	664	675	676
	ΔS	13	12	12	15
	ε	2.93	2.32	2.12	1.97
	Φ_F_	1.00	0.12	0.04	0.05
	Φ_Δ_	<0.01	<0.01	<0.01	<0.01
CFM	λ_abs_	638	647	657	658
	λ_em_	639	656	670	671
	ΔS	1	9	13	13
	ε	4.28	3.30	2.02	2.81
	Φ_F_	0.46	0.08	0.10	0.06
MeOH	λ_abs_	631	640	650	650
	λ_em_	644	652	661	663
	ΔS	13	12	11	13
	ε	4.22	3.03	1.74	2.85
	Φ_F_	0.16	0.02	0.01	0.01

**Table 2:** Estimated free binding energies (kcal/mol) of reference ligands (warfarin and ibuprofen) and *N*-propylbenzene indolenine-based squaraine dyes (**5**, **9**, **11**, and **14**) with human serum albumin (HSA) at Sudlow site I (2BXD), Sudlow site II (2BXG), and the full albumin.

Ligand		Estimated Free Binding Energy (kcal/mol)				
	Warfarin	Ibuprofen	Dye 5	Dye 9	Dye 11	Dye 14
Protein 2BXD Sudlow Site I	−8.79	–	−14.25	−12.40	−12.64	−15.57
Protein 2BXG Sudlow Site II	–	−7.44	−10.97	−10.27	−10.01	−12.59
Protein 2BXG Full Protein	–	–	−11.77	−10.09	−9.75	−13.84

**Table 3:** Photophysical properties of squaraine dyes **5**, **9**, **11**, and **14** in phosphate buffer (pH 7.4) in the absence and presence of human serum albumin (HSA). Reported parameters include the absorption maximum (λ_abs_), emission maximum (λ_em_), Stokes shift (ΔS), molar absorption coefficient (ε, ×10^5^ M^−1^·cm^−1^), and fluorescence quantum yield (Φ_F_).

Squaraine Dye	HSA Absence					HSA Presence			
	λ_abs_	λ_em_	ΔS	ε	Φ_F_	λ_abs_	λ_em_	ΔS	Φ_F_
**5**	605	696	91	0.89	0.02	604	649	45	0.20
**9**	605	642	37	0.58	0.00	651	659	8	0.07
**11**	655	ND	–	0.71	0.01	652	662	10	0.04
**14**	672	ND	–	0.67	0.00	663	667	4	0.01

**Table 4:** Fluorescence parameters describing squaraine-albumin interactions for dyes **5**, **9**, **11**, and **14**. Reported values include the fluorescence intensity without protein (F_0_), the fluorescence intensity at the highest protein concentration (F), their ratio (F/F_0_), sensitivity (S), quantification limit (QL), detection limit (DL), and binding constant (K_β_).

Squaraine Dye	F_0_ (a.u.)	Squaraine-Protein Complex Fluorescence Properties					
		F (a.u.)	F/F_0_	S (nM)	QL (nM)	DL (nM)	K_b_ (M^−1^)
**5**	11.46	680.15	59.35	2.09 × 10^5^	886	266	2.34 × 10^5^
**9**	9.30	917.53	98.66	3.18 × 10^5^	1079	324	6.23 × 10^5^
**11**	1.00	360.94	360.94	1.19 × 10^5^	1401	420	2.30 × 10^6^
**14**	4.12	115.89	28.13	3.9 × 10^4^	1203	361	3.75 × 10^5^

**Table 5:** Half-maximal inhibitory concentration (IC_50_ in μM) and their determination coefficient (r^2^) of *N*-propylbenzene indolenine-based squaraine dyes **5**, **9**, **11**, and **14** against normal human dermal fibroblasts (NHDF) and prostate adenocarcinoma (PC-3) cell lines under dark and light treatments. Photodynamic ratio (PTR) and tumor selectivity ratio (TSR) were also calculated. ND indicates values that were not determined.

		Squaraine Dyes							
Cell Line		5		9		11		14	
		IC_50_	r^2^	IC_50_	r^2^	IC_50_	r^2^	IC_50_	r^2^
NHDF	Dark	>10	–	6.803	0.95	6.573	0.93	>10	–
	Light	7.406	0.97	3.794	0.95	3.239	0.91	>10	–
	PTR	>1.4		1.8		2.0		ND	
PC-3	Dark	>10	–	1.210	1.00	1.442	0.96	>10	–
	Light	0.898	0.98	0.093	0.99	0.09	0.93	>10	–
	PTR	>11.1		13.0		16.0		ND	
	TSR	8.2		40.8		36.0		ND	

**Table 6:** Excitation wavelengths and excitation/emission slit settings employed for fluorescence measurements of squaraine-protein binding.

Squaraine Dye	Excitation Wavelength (nm)	Excitation/Emission Slits (nm)
**5**	600	10/10
**9**	600	10/10
**11**	600	5/5
**13**	600	10/10

## Data Availability

The original contributions presented in this study are included in the article/supplementary material. Further inquiries can be directed to the corresponding author(s).

## Supplementary Materials

The following supporting information can be downloaded at: https://www.mdpi.com/article/10.3390/ijms262210989/s1.
