# Supplementary material for: Fluorescent Albumin-Binding N-Propylbenzene Indolenine-Based Squaraines as Potential Candidates for Prostate Cancer Photodynamic Therapy Photosensitizers
Source: Int J Mol Sci. 2025 Nov 13;26(22):10989. doi: 10.3390/ijms262210989 (PMC12652113; doi:10.3390/ijms262210989)
Supplement: Supplementary file 1 [file ijms-26-10989-s001.zip › ijms-3954417-supplementary.pdf]

International Journal of Molecular Sciences

MDPI

Supplementary material

Fluorescent albumin-binding *N*-propylbenzene indolenine-based squaraines as potential candidates for prostate cancer photodynamic therapy photosensitizers

Catarina Costa [1,†], Eurico Lima [1,2,†], Maria Vaz [1], Octávio Ferreira [2], Renato E. Boto [3], Paulo Almeida [3], José R. Fernandes [1], Samuel M. Silvestre [3,*] and Lucinda V. Reis [2,*]

[1] CQ-VR—Chemistry Centre of Vila Real, University of Trás-os-Montes and Alto Douro, Quinta de Prados, 5001-801 Vila Real, Portugal;
[2] RISE-Health, Faculty of Health Sciences, University of Beira Interior, Avenida Infante D. Henrique, 6201-506, Covilhã, Portugal;
[3] RISE-Health, Faculty of Sciences, University of Beira Interior, Rua Marquês d'Ávila e Bolama, 6201-001, Covilhã, Portugal.
* Correspondence: sms@ubi.pt (S.M.S.); lucinda.reis@utad.pt (L.V.R.)
† Both authors can be considered first author since they contributed equally to this work.

International Journal of *Molecular Sciences*

MDPI

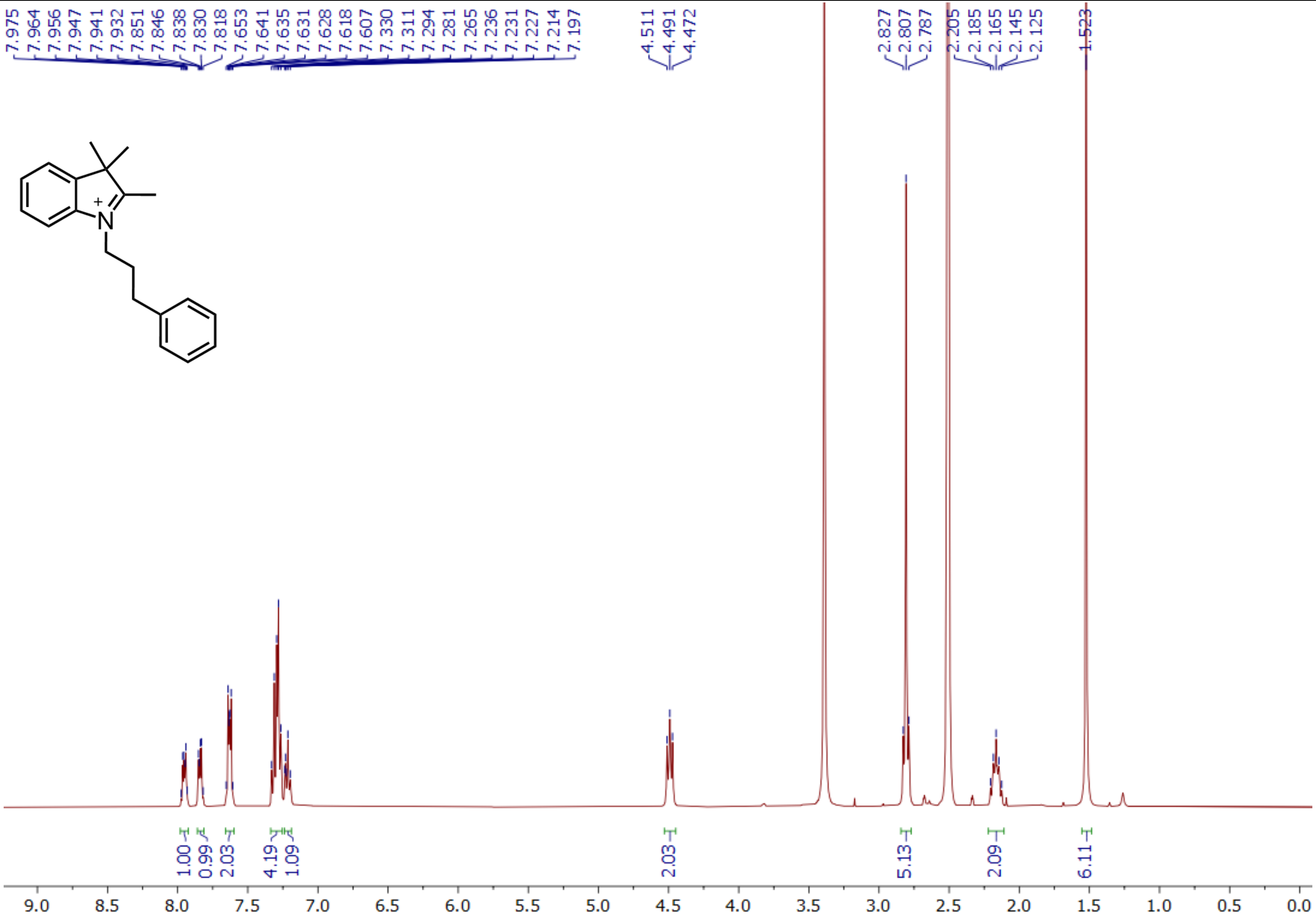

Figure S1. ^{1}H NMR spectrum of *N*-propylbenzene indolenine-based quaternary ammonium salt **3** (400.13 MHz, DMSO-d_6, ppm).

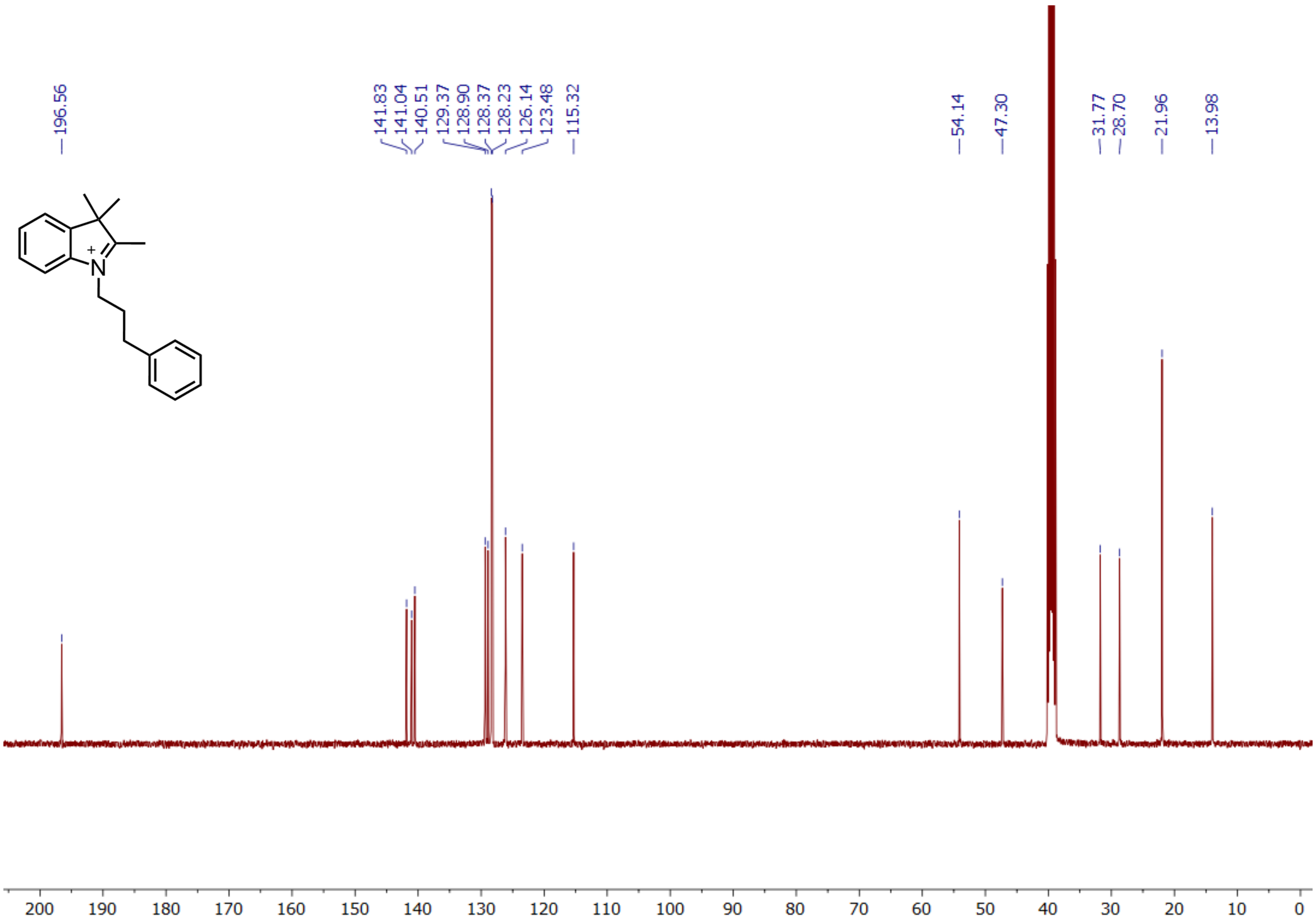

Figure S2. ^{13}C NMR spectrum of *N*-propylbenzene indolenine-based quaternary ammonium salt **3** (100.62 MHz, DMSO-d_6, ppm).

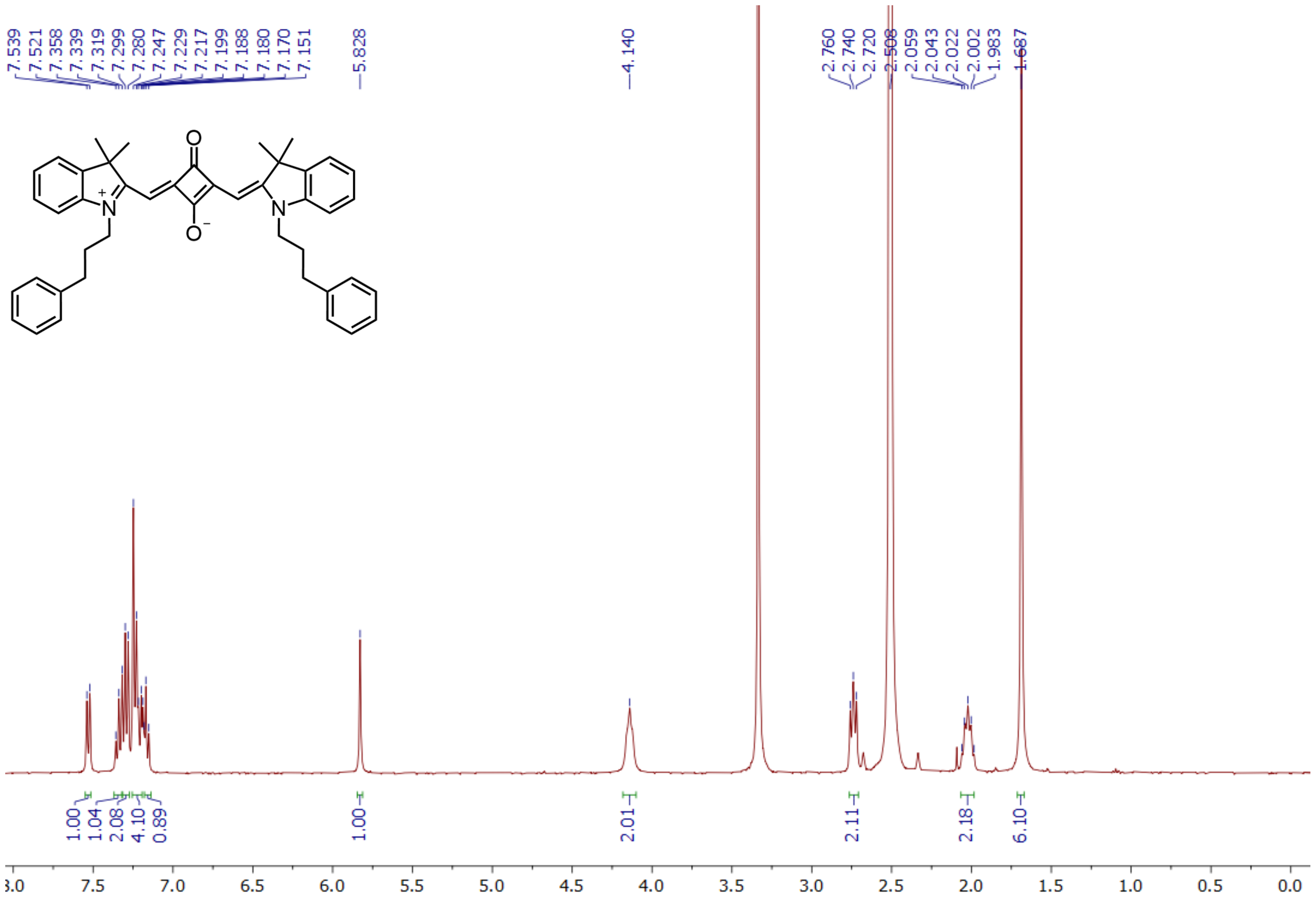

Figure S3. ¹H NMR spectrum of *N*-propylbenzene indolenine-based unsubstituted squaraine dye **5** (400.13 MHz, DMSO-*d*₆, ppm).

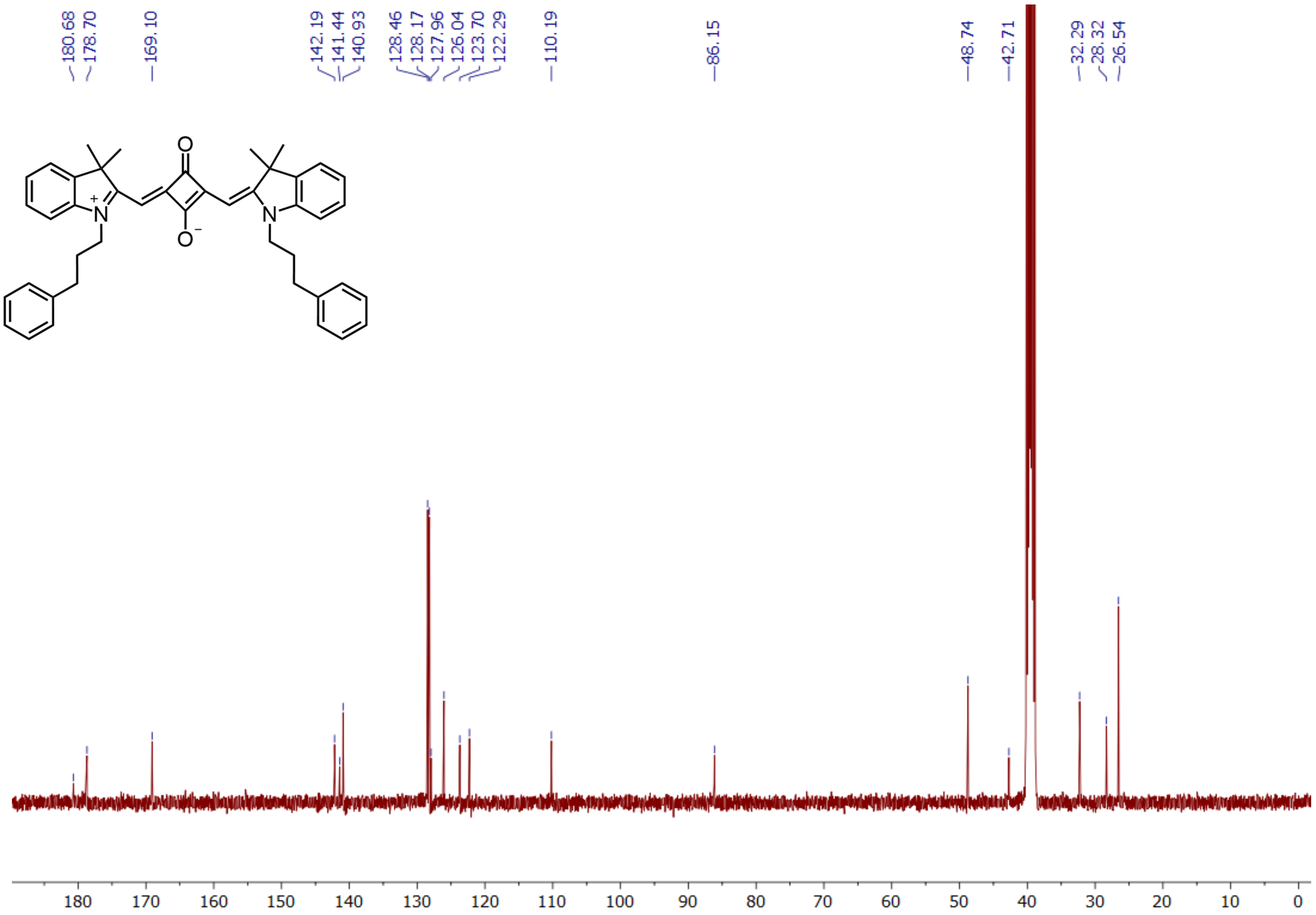

Figure S4. ^{13}C NMR spectrum of *N*-propylbenzene indolenine-based unsubstituted squaraine dye **5** (100.62 MHz, DMSO-d_6, ppm).

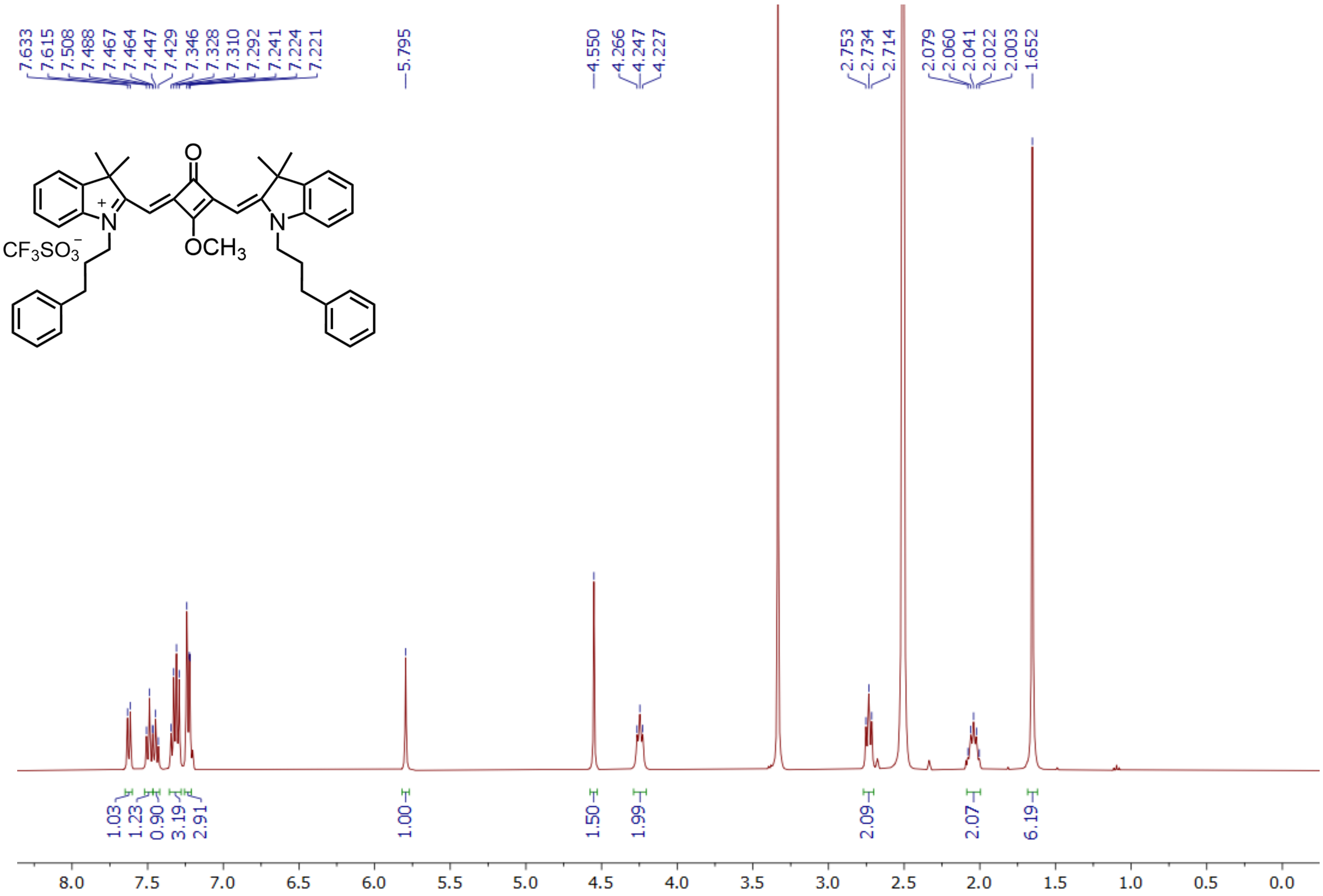

Figure S5. ^{1}H NMR spectrum of *N*-propylbenzene indolenine-based *O*-methylated squaraine dye **7** (400.13 MHz, DMSO-*d*$_6$, ppm).

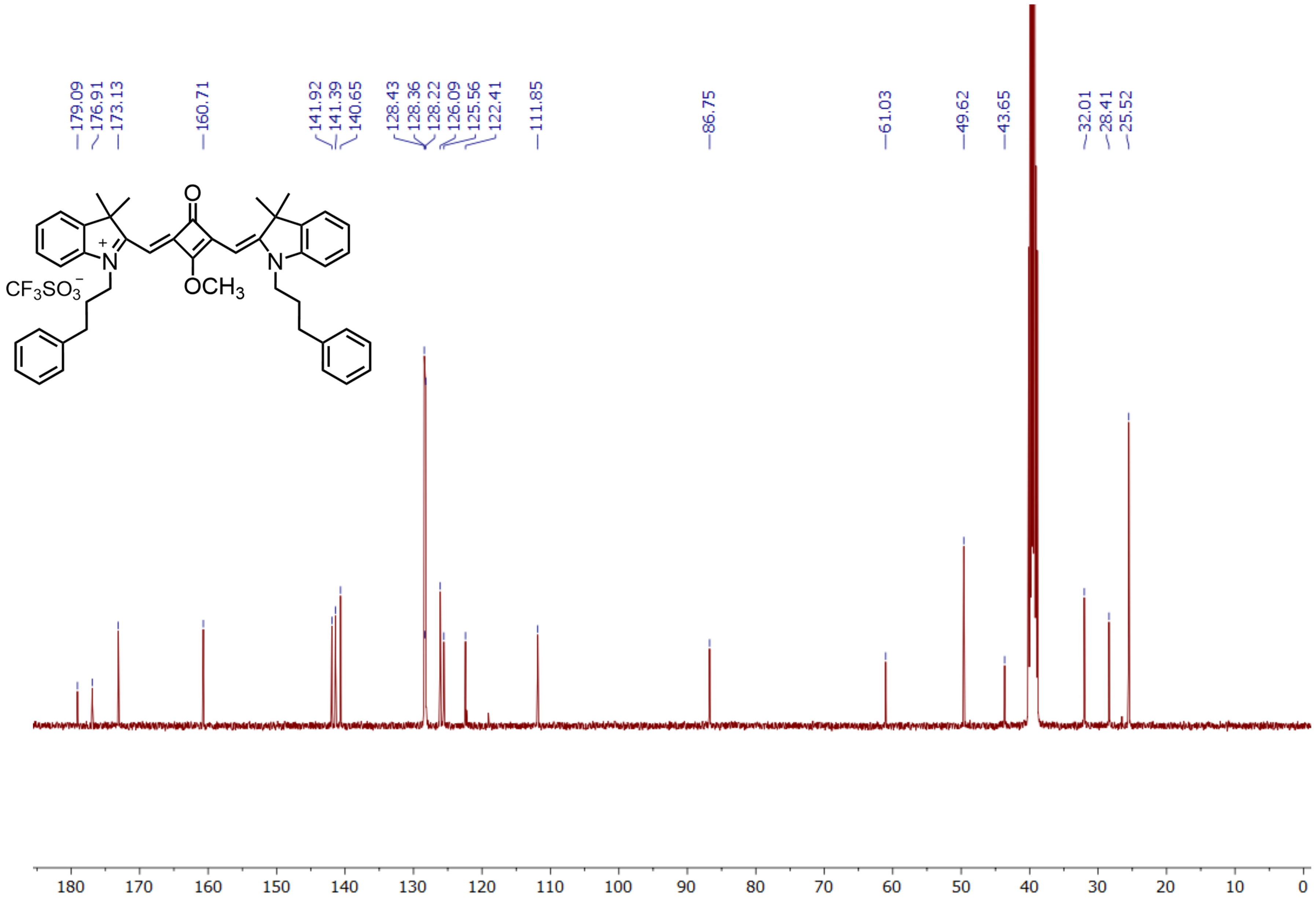

Figure S6. ^{13}C NMR spectrum of *N*-propylbenzene indolenine-based *O*-methylated squaraine dye **7** (100.62 MHz, DMSO-*d*6, ppm).

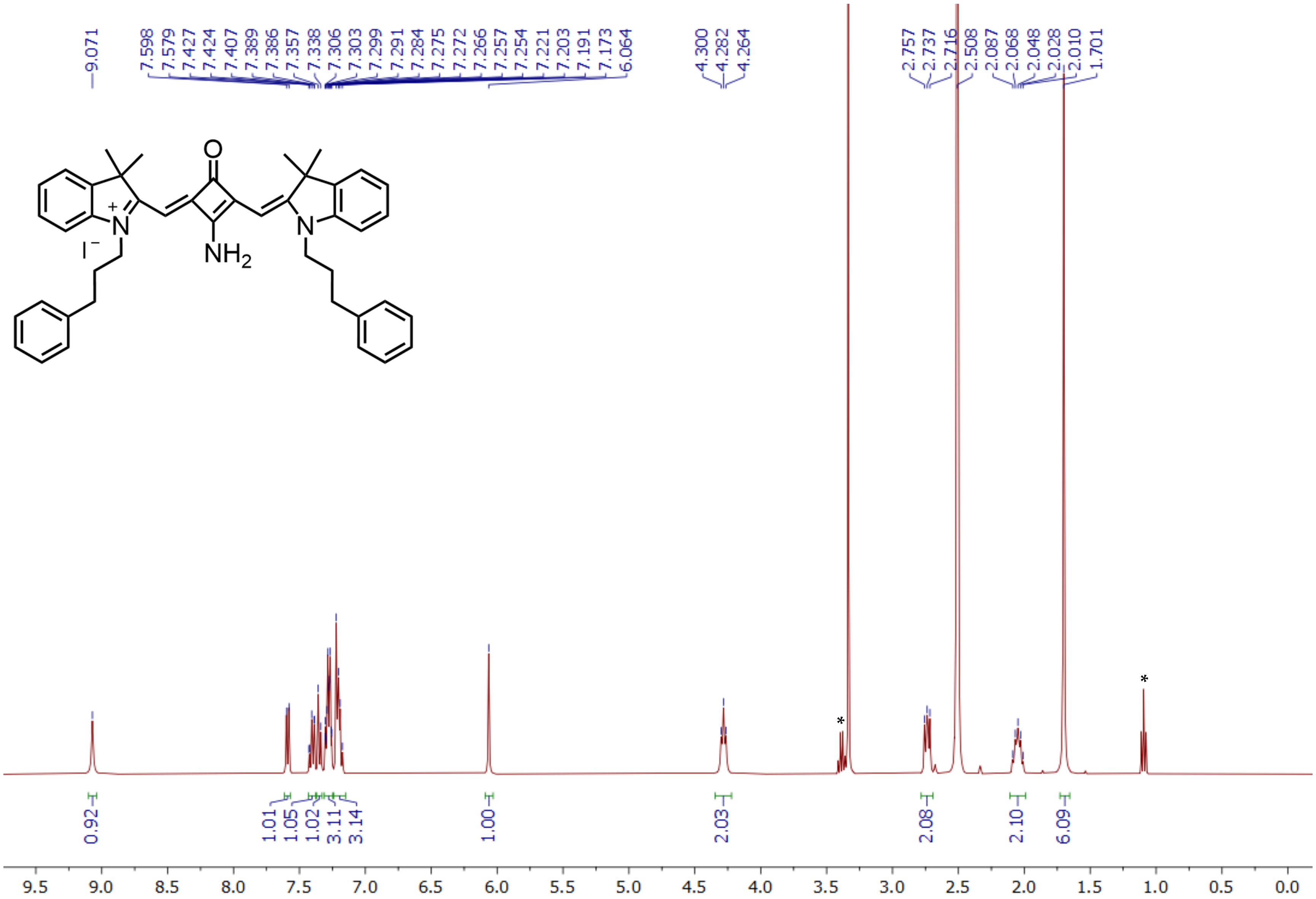

Figure S7. ^{1}H NMR spectrum of *N*-propylbenzene indolenine-based amino-bearing squaraine dye **9** (400.13 MHz, DMSO-d_6, ppm). *Residual solvent peaks

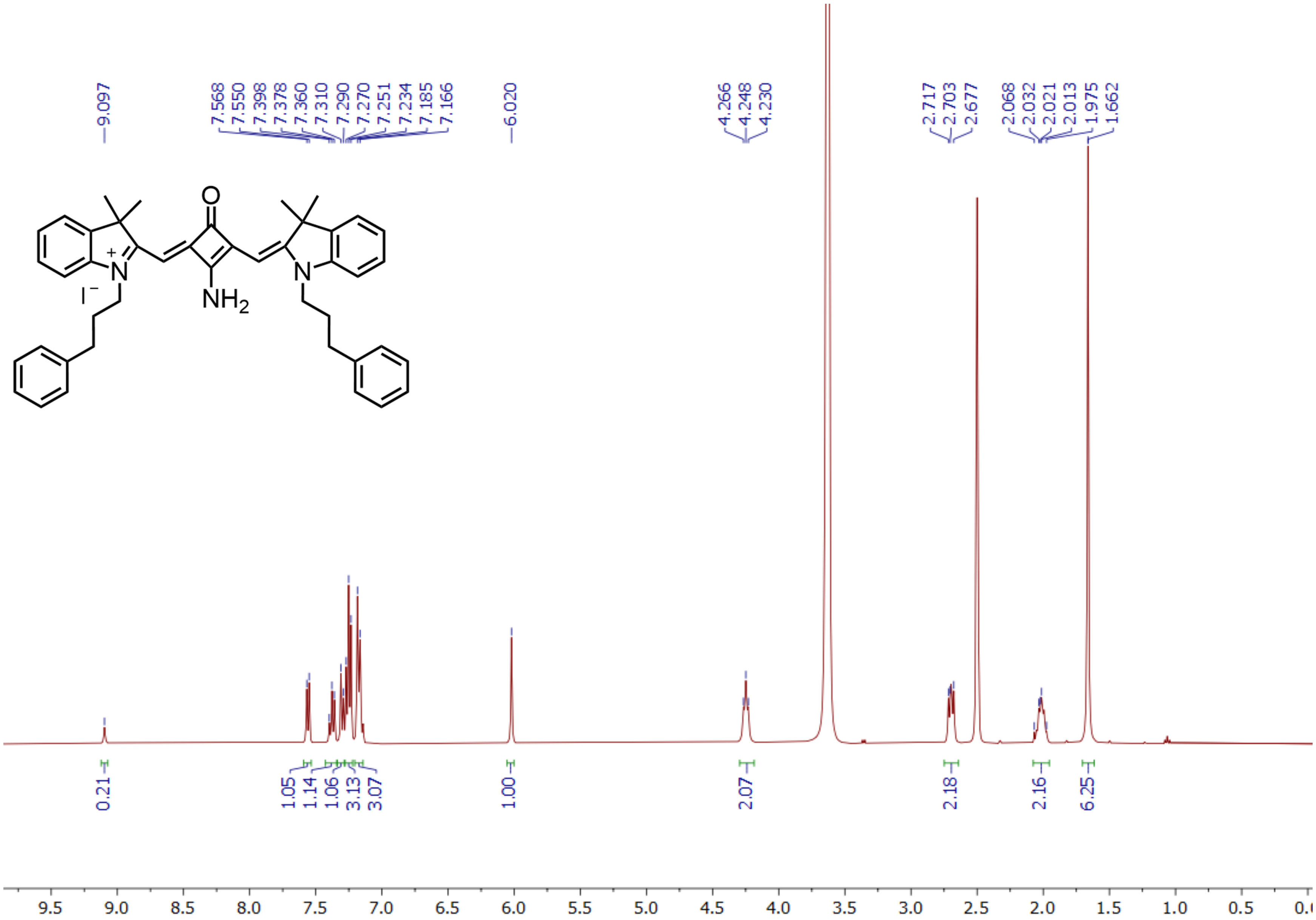

Figure S8. ^{1}H NMR spectrum of *N*-propylbenzene indolenine-based amino-bearing squaraine dye **9** (400.13 MHz, DMSO-d_6+ D_2O, ppm).

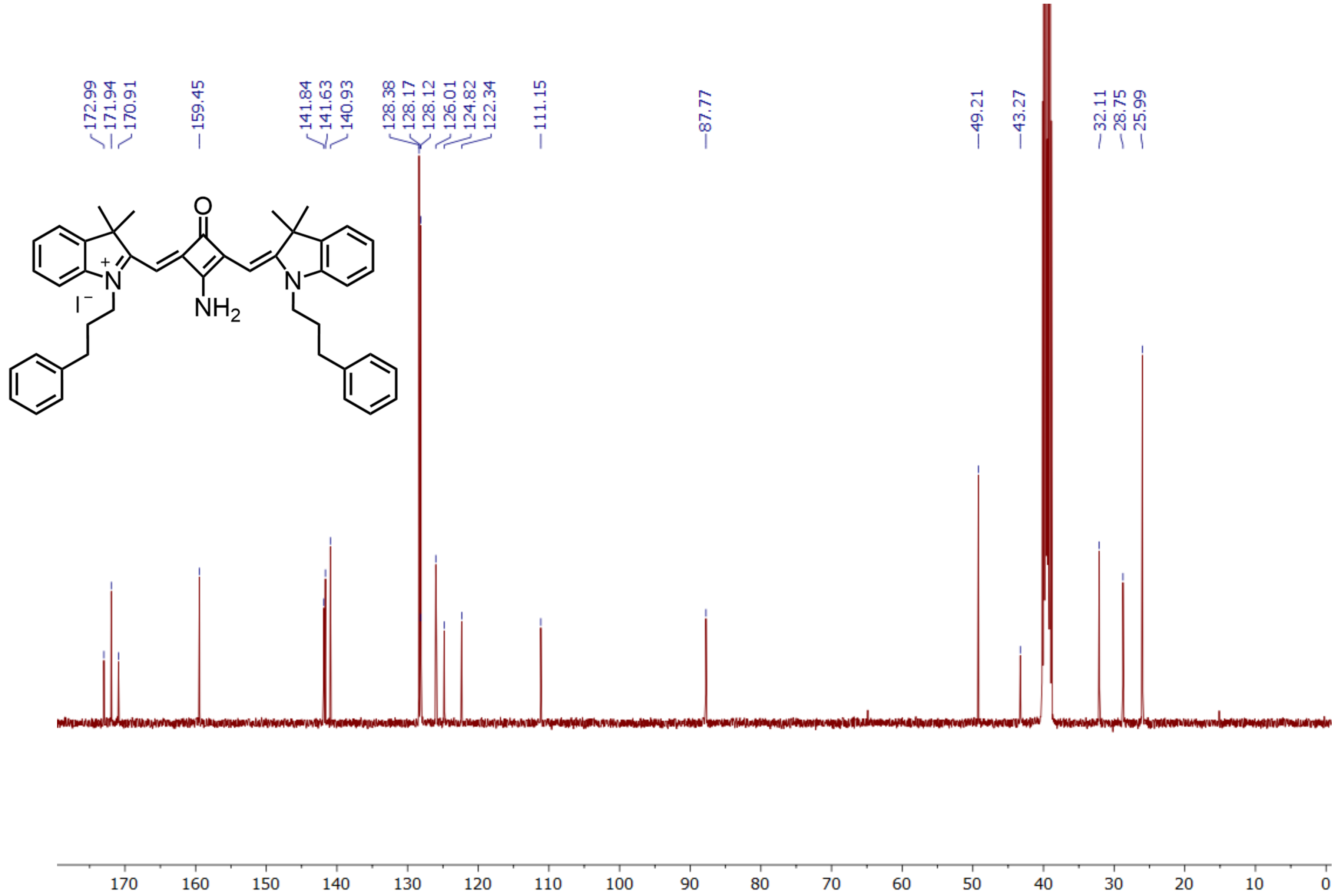

Figure S9. ^{13}C NMR spectrum of *N*-propylbenzene indolenine-based amino-bearing squaraine dye **9** (100.62 MHz, DMSO-d_6, ppm).

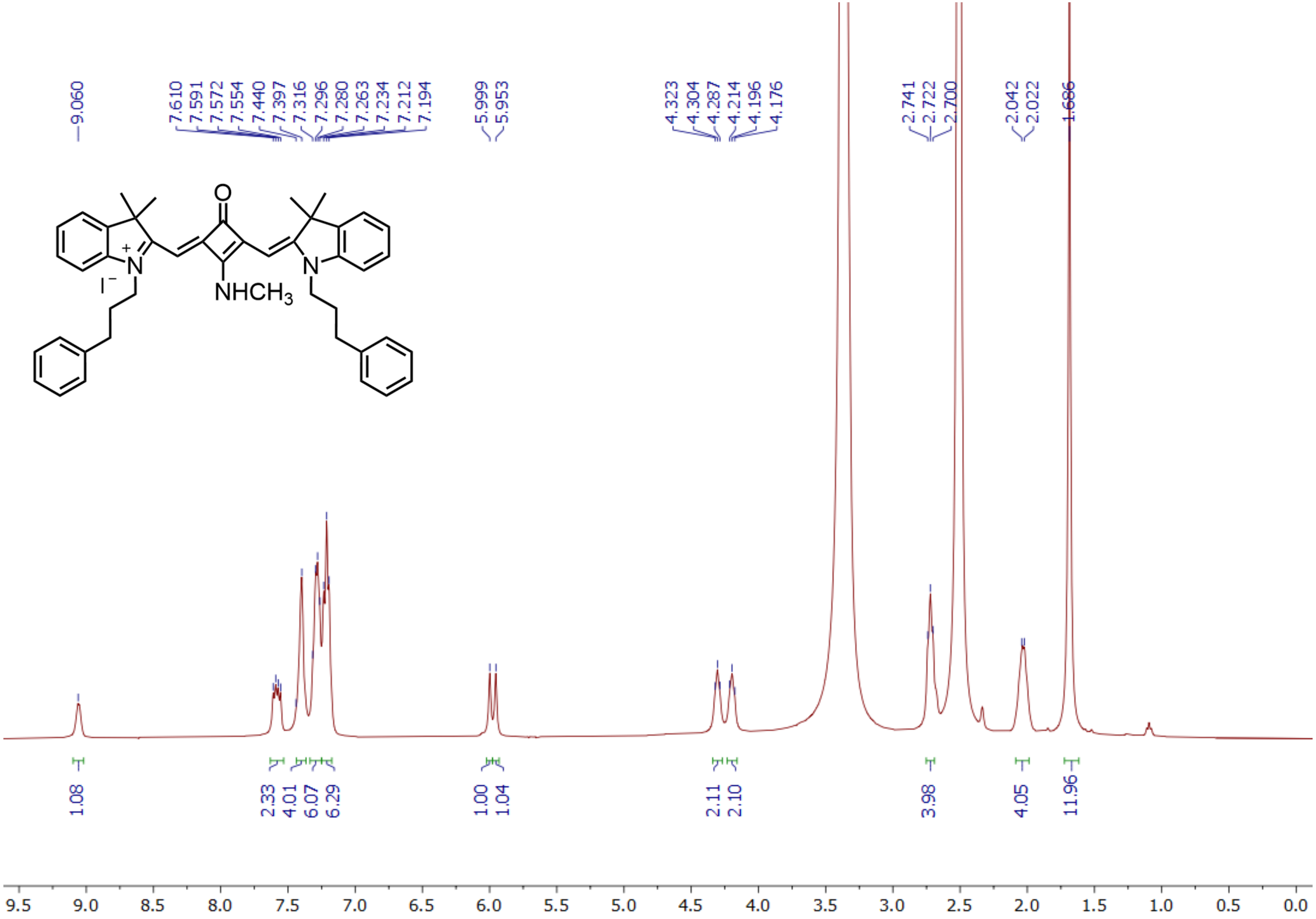

Figure S10. ^{1}H NMR spectrum of *N*-propylbenzene indolenine-based methylamino-bearing squaraine dye **11** (400.13 MHz, DMSO-d_6, ppm).

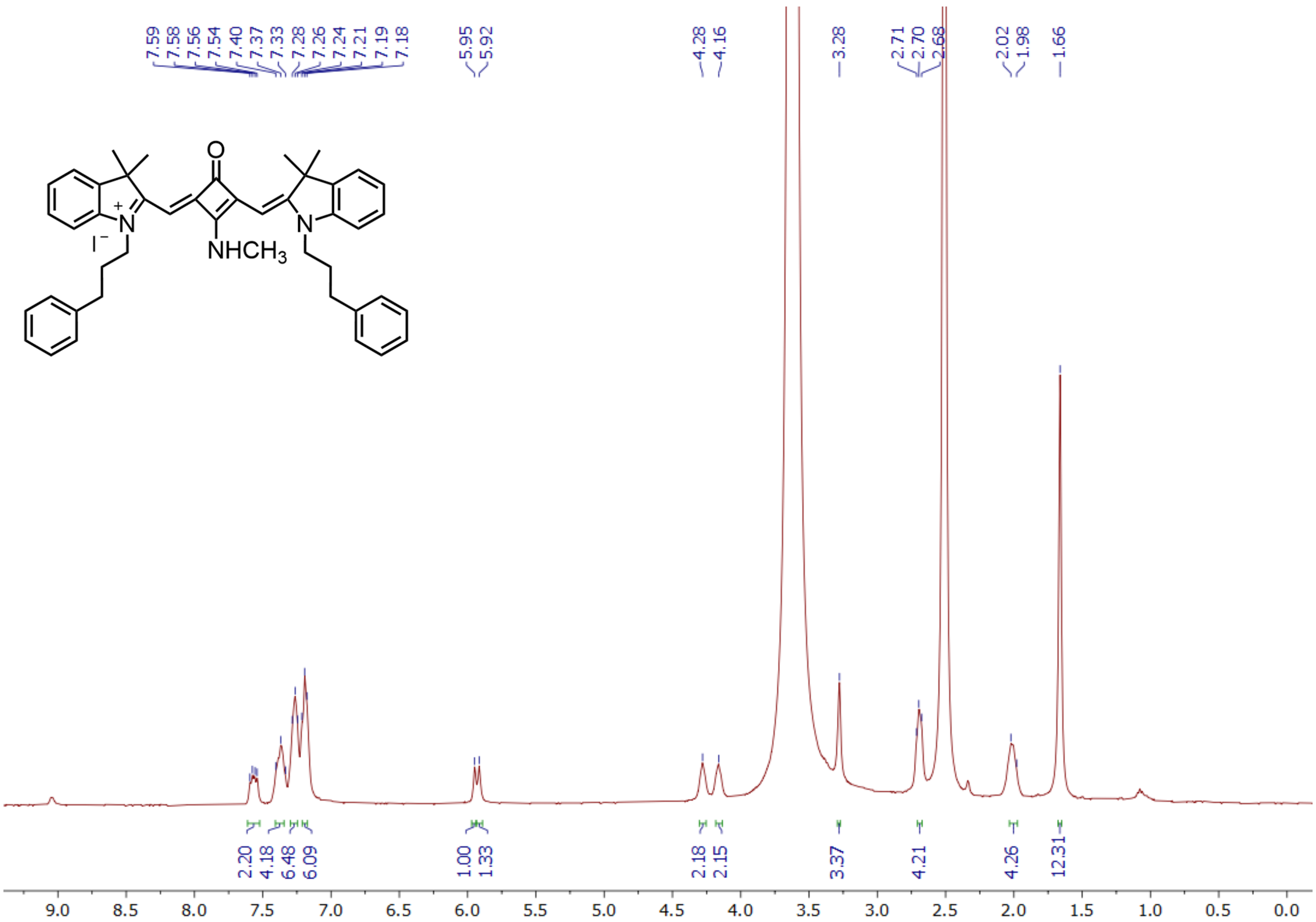

35

Figure S11. ^{1}H NMR spectrum of *N*-propylbenzene indolenine-based methylamino-bearing squaraine dye **11** (400.13 MHz, DMSO-d_6 + D_2O, ppm). 36

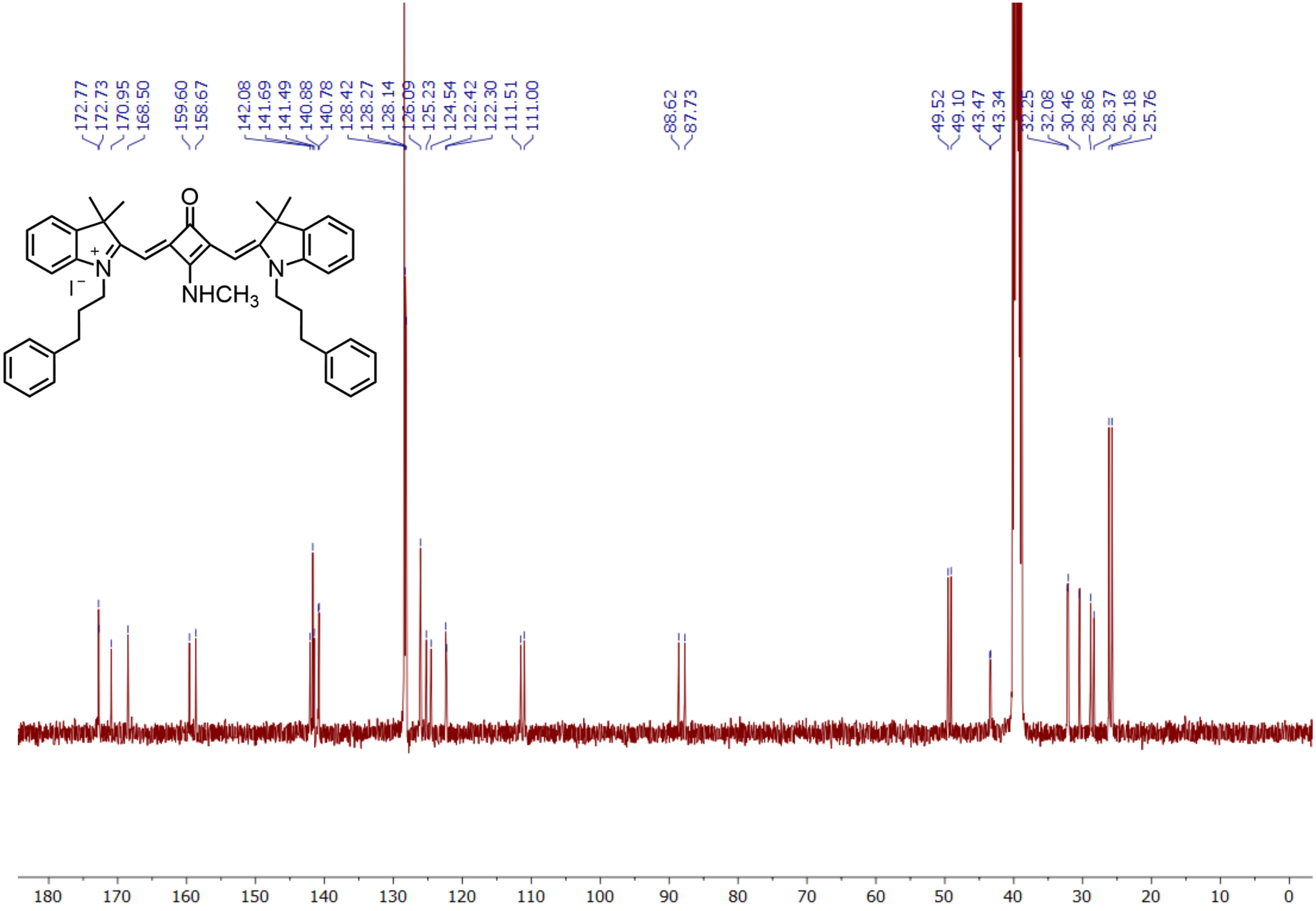

37

Figure S12. ^{13}C NMR spectrum of *N*-propylbenzene indolenine-based methylamino-bearing squaraine dye **11** (100.62 MHz, DMSO-d_6, ppm). 38

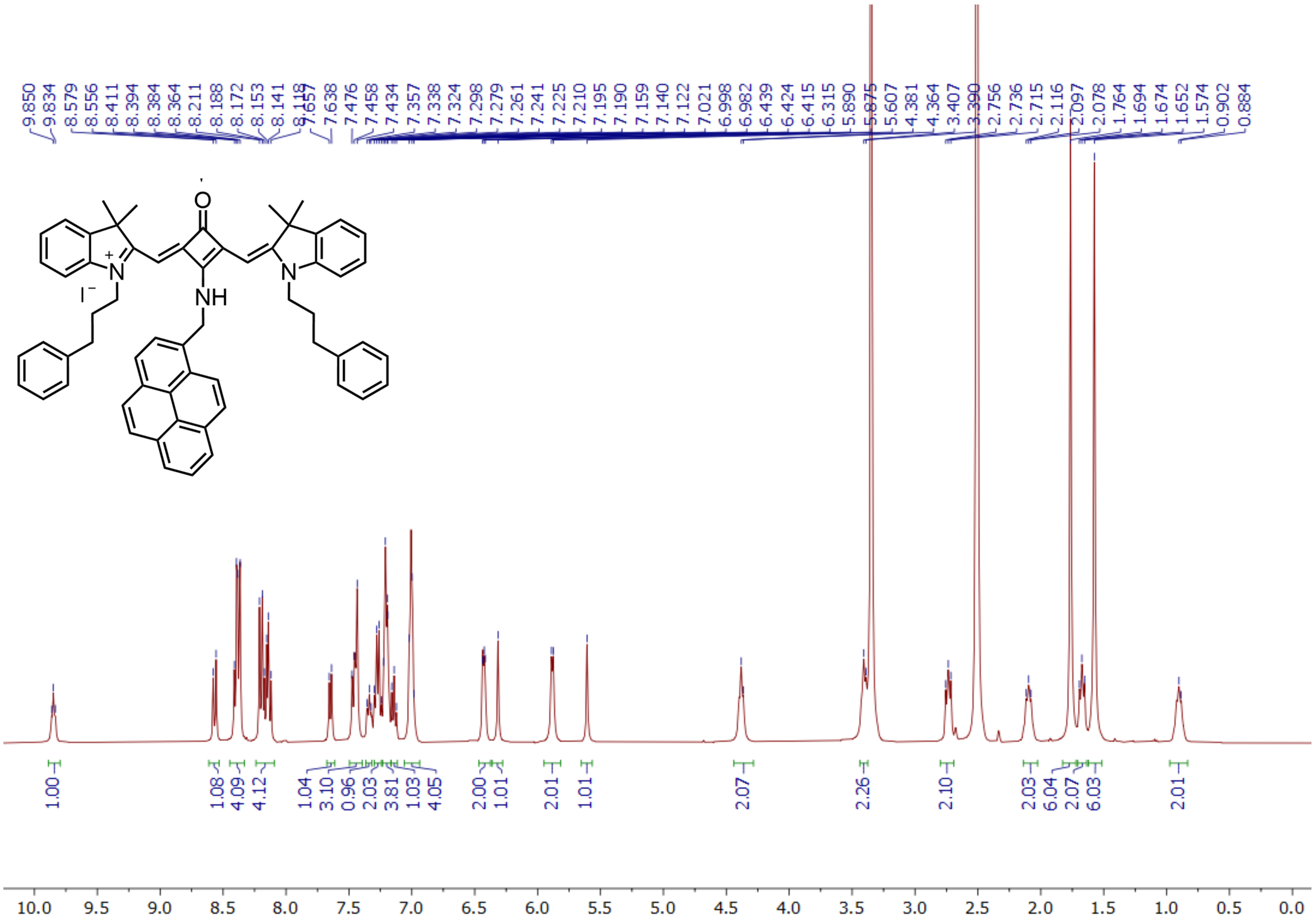

Figure S13. ^{1}H NMR spectrum of *N*-propylbenzene indolenine-based aminomethylpyrene-bearing squaraine dye **14** (400.13 MHz, DMSO-d_6, ppm).

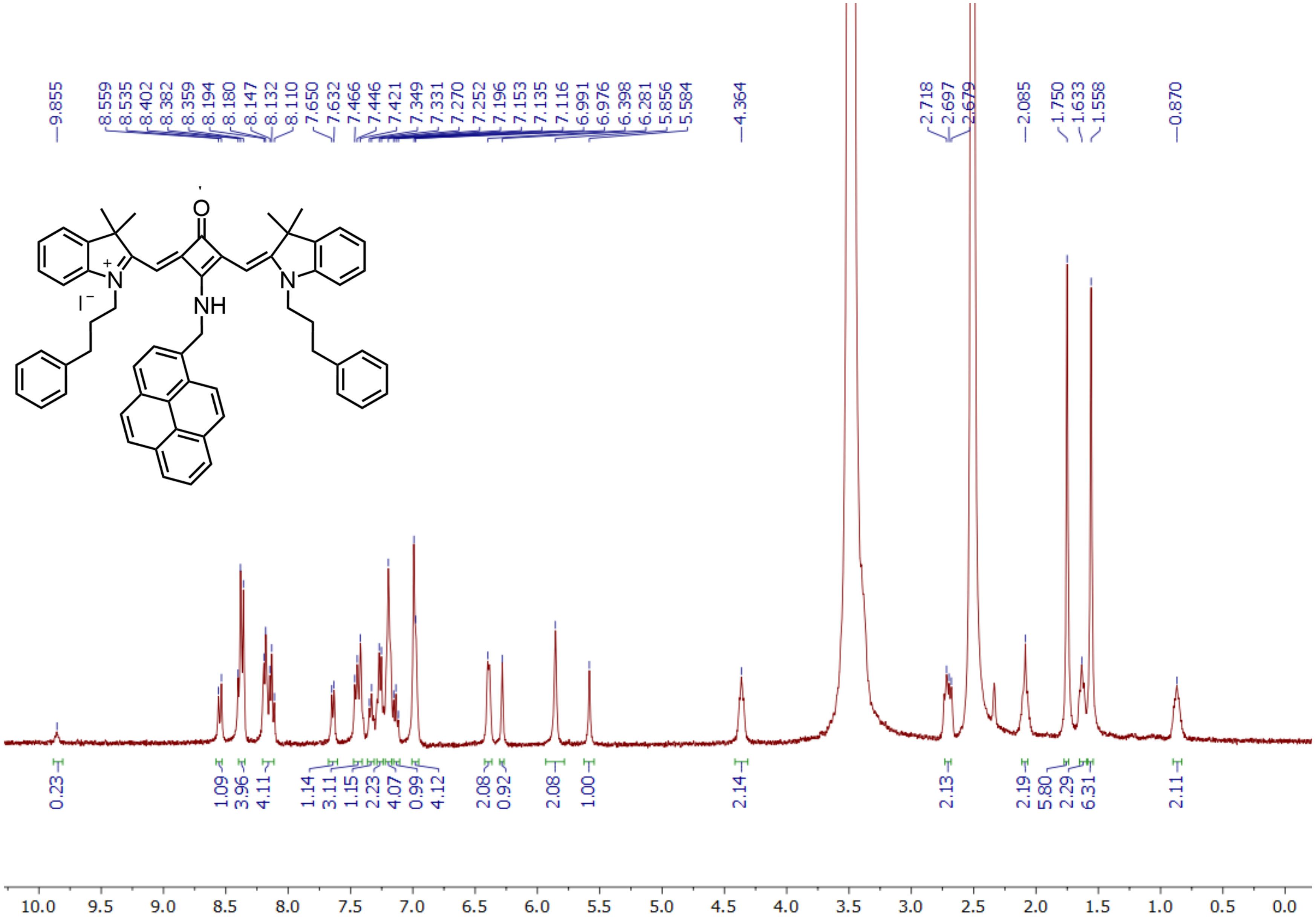

Figure S14. ^{1}H NMR spectrum of *N*-propylbenzene indolenine-based aminomethylpyrene-bearing squaraine dye **14** (400.13 MHz, DMSO-d_6 + D_2O, ppm).

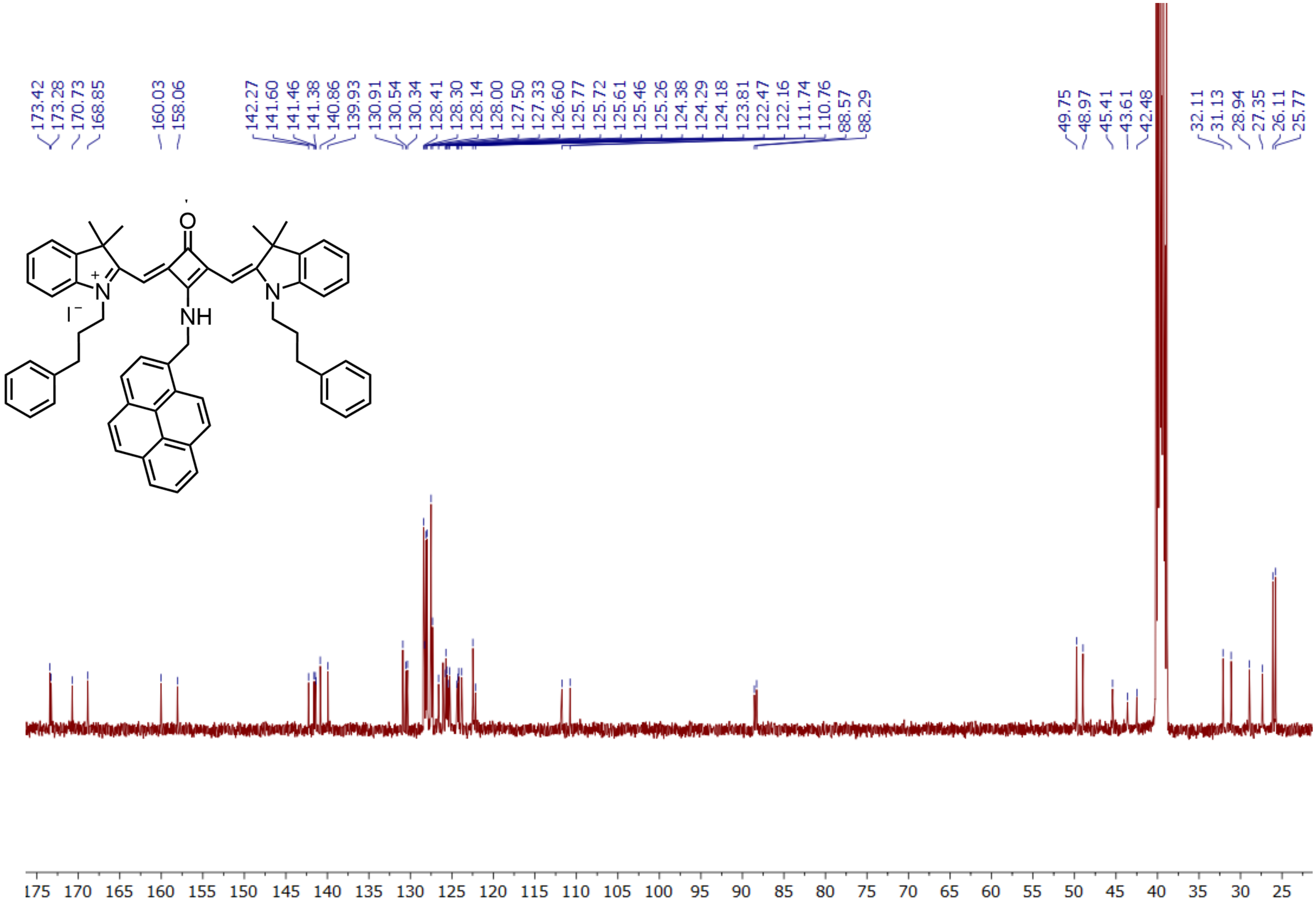

43

Figure S15. ^{13}C NMR spectrum of *N*-propylbenzene indolenine-based methylamino-bearing squaraine dye **14** (100.62 MHz, DMSO-d_6, ppm). 44

International Journal of *Molecular Sciences*

MDPI

Table S1. Predicted interactions list of warfarin and *N*-propylbenzene indolenine-based squaraine dyes **5**, **9**, **11** and **14** in Sudlow site I of human serum albumin.

**Protein: 2BXD (Sudlow's Site I)**		**Ligand**				
		**Warfarin**	**5**	**9**	**11**	**14**
**Interactions**	**Classic H-Bonds**	ARG218<br>ARG222	LYS195	ARG218<br>ARG222	LYS195	ARG218<br>ARG222
	**Non-classic H-Bonds[a]**	LYS199	ARG218	–	ARG218	ARG218
	**Van der Waals**	–	–	–	–	–
	**Electrostatics[b]**	LYS195<br>ARG222	LYS199<br>ARG218<br>ASP451	LYS195<br>LYS199	LYS199<br>ARG218<br>ASP451	LYS195<br>LYS199
	**Miscellaneous**	CYS448[d]	CYS448[d]	–	CYS448[d]	–
	**Hydrophobic**	LYS195<br>LEU198<br>TRP214<br>LEU219<br>LEU238<br>ALA291<br>VAL455	LYS195<br>LEU198<br>LYS199<br>TRP214<br>ALA215<br>ARG218<br>LEU219<br>LEU238<br>ALA291<br>VAL343<br>ASP451	LYS195<br>LEU198<br>LYS199<br>TRP214<br>ALA291<br>PRO447<br>CYS448<br>VAl455<br>LEU481	LYS195<br>LEU198<br>LYS199<br>TRP214<br>ALA215<br>ARG218<br>LEU219<br>LEU238<br>ALA291<br>VAL343<br>ASP451	ALA194<br>LYS195<br>LEU198<br>PHE211<br>TRP214<br>ALA215<br>LEU238<br>ALA291<br>PRO447<br>CYS448<br>TYR452<br>VAL455
	**Unfavorable[c]**	–	–	LYS199<br>ARG218	–	–

[a] Non-classical H-bonds: carbon-hydrogen; π-donor hydrogen bond;

[b] Electrostatic interactions: π-cation; π-anion; attractive charge; salt bridge;

[c] Unfavorable interactions: charge repulsion;

[d] π-Sulfur interactions.

International Journal of Molecular Sciences

MDPI

Table S2. Predicted interactions list of ibuprofen and *N*-propylbenzene indolenine-based squaraine dyes **5**, **9**, **11** and **14** in Sudlow site II of human serum albumin.

**Protein: 2BXG (Sudlow's Site II)**		**Ligand**				
		**Ibuprofen**	**5**	**9**	**11**	**14**
**Interactions**	**Classical H-Bonds**	ARG410<br>TYR411	ASN483	ASN483	–	–
	**Non-Classical H-Bonds**[a]	–	–	–	GLU383	–
	**Van der Waals**	–	–	–	–	–
	**Electrostatics**[b]	–	–	–	–	GLU383
	**Miscellaneous**	–	–	–	–	CYS448[d]
	**Hydrophobic**	ILE388<br>CYS392<br>LEU407<br>LEU430<br>VAL433<br>CYS437<br>CYS438<br>ALA449<br>LEU453	LYS351<br>THR352<br>CYS476<br>PRO379<br>LEU380<br>PRO486<br>CYS487<br>ALA490	ARG348<br>LYS351<br>CYS476<br>PRO379<br>LEU380<br>ARG484<br>PRO486<br>CYS487<br>ALA490	ARG348<br>LYS351<br>PRO379<br>LEU380<br>PRO486<br>CYS487<br>ALA490	ARG348<br>LYS351<br>GLU376<br>PRO379<br>LEU380<br>CYS448<br>PRO486<br>SER489<br>ALA490
	**Unfavorable**[c]	–	–	–	–	–

[a] Non-classical H-bonds: carbon-hydrogen; π-donor hydrogen bond;

[b] Electrostatic interactions: π-cation; π-anion; attractive charge; salt bridge;

[c] Unfavorable interactions: charge repulsion;

[d] π-Sulfur interactions;

[e] π-Lone pair interactions.

Table S3. Predicted interactions list of *N*-propylbenzene indolenine-based squaraine dyes **5**, **9**, **11** and **14** considering full human serum albumin.

**Protein: 2BXG (All Protein)**		**Ligand**			
		**5**	**9**	**11**	**14**
**Interactions**	**Classical H-Bonds**	–	–	GLU520	–
	**Non-Classical H-Bonds**[a]	–	–	LYS524<br>THR527	ARG145
	**Van der Waals**	–	–	–	–
	**Electrostatics**[b]	ARG117	–	GLU425	ARG145<br>ARG186
	**Miscellaneous**	–	MET123[d]	–	–
	**Hydrophobic**	LEU115<br>ARG117<br>PRO118<br>MET123<br>ALA126<br>PHE134<br>LYS137<br>ILE142<br>LEU182<br>ARG186	PRO35<br>PHE36<br>PRO113<br>LEU115<br>PRO118<br>ALA126<br>PHE134<br>LYS137	PHE507<br>ILE513<br>ILE523<br>LYS524<br>ALA528	PRO110<br>ARG114<br>LEU115<br>VAL116<br>LYS137<br>TYR138<br>ILE142<br>ARG145<br>LEU182<br>ARG186
	**Unfavorable**[c]	–	–	–	HIS146

[a] Non-classical H-bonds: carbon-hydrogen; π-donor hydrogen bond;

[b] Electrostatic interactions: π-cation; π-anion; attractive charge; salt bridge;

[c] Unfavorable interactions: charge repulsion;

[d] π-Sulfur interactions.
